# Amide additives enhance the understanding of kinetic reversibility in zinc anode stability using ultramicroelectrodes

**DOI:** 10.1039/d5sc06311f

**Published:** 2025-10-31

**Authors:** James H. Nguyen, Ashutosh Rana, Kudekallu Shiprath, Brajesh R. Bhagat, Saptarshi Paul, Shaonsikta Chatterjee, Newton Roy, Ishita Das, Bidisa Das, Abhik Banerjee, Jeffrey E. Dick

**Affiliations:** a Department of Chemistry, Purdue University West Lafayette IN 47907 USA jdick@purdue.edu; b Research Institute for Sustainable Energy, Center for Research and Education in Science and Technology (TCG-CREST) Salt Lake Kolkata 700091 India abhik.banerjee@tcgcrest.org bidisa.das@tcgcrest.org; c Academy of Scientific and Innovative Research (AcSIR) Ghaziabad 201002 India; d Elmore Family School of Electrical and Computer Engineering, Purdue University West Lafayette IN 47907 USA

## Abstract

Aqueous zinc metal batteries (AZMBs) offer safety and sustainability but face challenges from hydrogen evolution, corrosion, and dendrite formation in mildly acidic electrolytes. Electrolyte additives improve anode stability by modifying interfacial chemistry through surface adsorption or altering zinc ion solvation. However, the mechanisms by which trace amounts of additives, often less than one percent of total ions, yield large performance improvements remain unclear. This suggests highly specific interfacial effects that require deeper investigation of charge transfer kinetics and interfacial resistances. Using fast scan voltammetry on ultramicroelectrodes (UMEs), we show that additives affect both the exchange current density and kinetic reversibility, a parameter reflecting the steady-state regime at high scan rates. We propose kinetic reversibility as a complementary metric to evaluate anode stability. Three amide-based additives, hexamethylphosphoramide, trimethylphosphoramide, and phosphoramide differing only in methyl substitution on the amide nitrogen, serve as model systems to study how molecular structure influences solvation, adsorption, and plating behavior. Electroanalysis on UMEs, supported by density functional theory, reveals the interplay of kinetics and interfacial chemistry. Galvanostatic cycling and morphological studies validate these findings. This work provides mechanistic insight and introduces kinetic reversibility as a valuable design criterion for stable zinc metal anodes.

## Abstract 2

With ultramicroelectrodes, we demonstrate how kinetic reversibility can be used alongside the conventionally employed exchange current density to offer a more comprehensive descriptor of anode stability.

## Graphical abstract

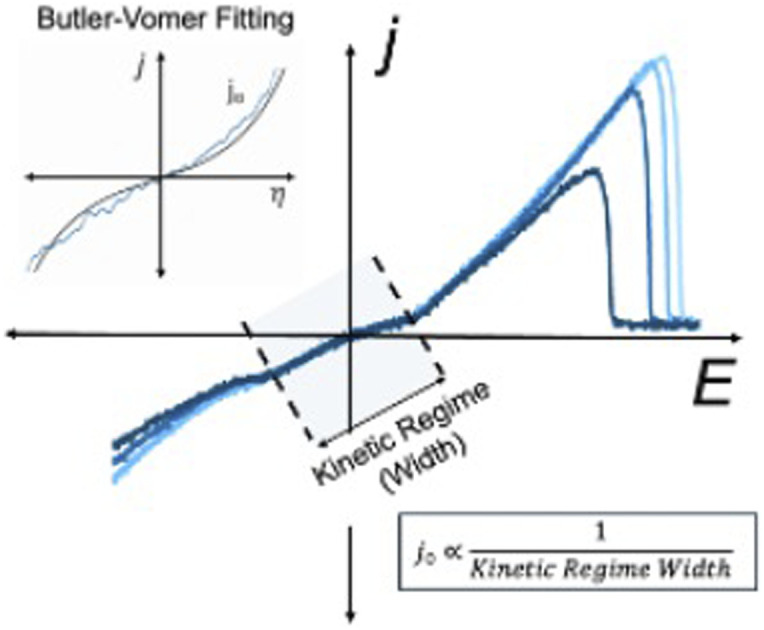

## Introduction

Aqueous zinc metal batteries (AZMBs) with Zn metal as the anode are emerging as a next-generation grid energy storage technology due to their combination of safety, sustainability, and electrochemical performance.^1–4^ Zn metal is abundant and an inexpensive that has a low redox potential of −0.76 V *versus* the standard hydrogen electrode.^5,6^ This makes Zn metal suitable as an anode material. Additionally, zinc exhibits a high theoretical capacity of 820 mA h g^−1^, significantly surpassing that of conventional lithium-ion battery anodes like graphite (372 mA h g^−1^).^7,8^ Lithium-ion systems typically rely on flammable organic electrolytes, whereas AZMBs often utilize aqueous electrolytes, making them inherently safer and environmentally friendly.^9^ Despite these advantages, AZMBs also face notable challenges. Much of the research in this field focuses on mildly acidic aqueous zinc electrolytes, which introduce intrinsic thermodynamic instability for the Zn metal anode.^10–12^ This instability gives rise to parasitic side reactions, including hydrogen evolution reaction (HER), zinc corrosion, and dendritic growth, all of which hinder the long-term performance and development of zinc-based batteries. In the literature, parasitic side reactions such as HER, zinc corrosion, and dendritic growth which impede the performance of AZMBs are often mitigated through strategies such as the incorporation of electrolyte additives, novel electrolyte formulations, and engineered current collector designs.^13–16^

Out of all the strategies, electrolyte additives are often typical in AZMB research because they provide a convenient, straightforward and tunable approach to address the drawbacks associated with AZMBs.^17–19^ Tuning the additives chemistry can improve zinc deposition, suppress dendrite formation, or mitigate side reactions like HER and passivation.^20,21^ The adjustment of the chemical structure or change in concentration of additives can enhance battery performance without significantly perturbing the native electrolyte system. As commonly reported in the field of AZMBs, additive chemistries with strong electron-donating capabilities often characterized by higher Gutmann donor numbers have been shown to significantly enhance the performance of AZMBs.^22–24^ These improvements are primarily attributed to modifications in solvation energetics, which, in turn, lead to slower electron transfer kinetics and suppression of parasitic side reactions.^25–27^ In addition to modulating solvation, such additive molecules often exhibit preferential binding to specific Zn crystal facets, offering corrosion protection and potentially influencing the overall kinetics of Zn electrodeposition.^28^ In terms of solvation modulation, these additives can alter the Zn^2+^ ion solvation shell by partially or fully replacing coordinated water molecules, thereby reshaping the local solvation structure. For instance, in a solvation structure study by Cao *et al.*, dimethyl sulfoxide (DMSO) was added where DMSO replaces the H_2_O in Zn^2+^ ion which inhibits the decomposition of solvated H_2_O.^26^ This adjustment leads to more controlled Zn^2+^ ion desolvation kinetics, promoting uniform nucleation and growth of zinc during electrodeposition. Additionally, by reducing the number of free water molecules in the solvation environment, these additives help suppress HER, a major parasitic side reaction in aqueous systems.^29,30^ Dai *et al.* used a bifunctional additive to not only affect the coordination environment around Zn^2+^ but also reconstruct the hydrogen bond network of water to improve liquid stability with ethylene glycol and sodium gluconate.^31^ The suppression of HER has commonly been reported in literature for the enhanced stability of Zn metal anode.^32,33^ On the other hand, the adsorption of additives onto the electrode surface can lead to the formation of a protective interfacial layer that mitigates parasitic side reactions such as HER by reducing the activity of solvated water.^34–39^ For instance, polyoxometalate can be added to the electrolyte, which binds with Zn^2+^ ions to produce a Zn film to regulate electrodeposition, significantly suppressing dendritic growth and suppress HER.^40^ Similar strategies are routinely observed in literature like: tetraalkylsufonamide in ZnCl_2_, polyvinylpyrrolidone in ZnSO_4_, vanillin in ZnSO_4_, and polyethylene oxide in ZnSO_4_.^32,41–43^ Additionally, certain works show that the electrolyte additives can also synergistically influence both these key processes. For instance, in an additive work by Rana *et al.*, imidazole not only affected the Zn solvation shell but also adsorbed onto the anode surface for suppressed side reactions and suppressed dendrite growth.^44^ Additives reported in literature like: tetraalkylsufonamide in ZnCl_2_, polyvinylpyrrolidone in ZnSO_4_, vanillin in ZnSO_4_, and polyethylene oxide in ZnSO_4_, which have been discussed above, have been shown to also work in a similar fashion.^32,41–43^ Furthermore, surface-adsorbed additives can reduce the activity of interfacial water molecules, thereby lowering proton availability and further suppressing HER at the electrode–electrolyte interface.^45–48^ The extent to which electrolyte additives stabilize zinc anodes through surface adsorption or solvation structure modulation remains unclear. Many additives likely engage in both mechanisms, with their relative contributions shifting under different electrochemical conditions. Yet, both are fundamentally interfacial processes. This raises an important but rarely addressed question: how can additives present at millimolar levels in molar zinc salt solutions; typically, less than 1% of total ionic content are able to deliver such significant improvements in performance and cyclability? This discrepancy suggests highly specific or cooperative interfacial effects that are not well understood. Despite widespread reports of additive-enhanced AZMB performance, the mechanistic basis remains largely speculative. A deeper investigation into charge transfer kinetics and interfacial resistances under realistic conditions is thus essential to understand how trace additives drive substantial gains in battery stability.

**Fig. 1:**
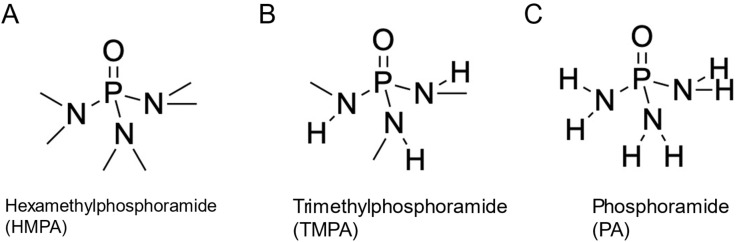
Phosphoramide additives used for their role on enhancing stability of zinc metal anode. (A) Hexamethylphosphoramide (HMPA). (B) Trimethylphosphoramide (TMPA). (C) Phosphoramide (PA).

Motivated by this, we deliberately designed a set of additive molecules with high electron-donating character, incorporating subtle variations in their substituent moieties surrounding the electron-donating centers. In this study, we systematically investigate the role of three amide-based additives hexamethylphosphoramide (HMPA), trimethylphosphoramide (TMPA), and phosphoramide (PA) in enhancing the stability of the zinc metal anode in a 1 M Zn(OTf)_2_ aqueous electrolyte (see synthesis conditions in SI) which are shown in Fig. 1. The amide additives were selected due to similar functional groups, differing only in the number of methyl substituents on the amide nitrogen atoms. HMPA, shown in Fig. 1A, contains six methyl substituents, two on each of the three amide nitrogen atoms. In contrast, TMPA (Fig. 1B) contains three methyl groups, with one methyl substituent on each nitrogen. The corresponding NMRs for TMPA can be shown in Fig. S1. Lastly, PA, shown in Fig. 1C, has no methyl groups and instead features only hydrogen atoms on the amide nitrogen atoms. The NMR for PA can be shown in Fig. S2. Subtle variations in additive molecular structure provide a unique platform to investigate how specific functional groups influence interfacial and solvation stabilization, thereby altering charge transfer kinetics. We evaluate these effects on zinc electrodeposition using ultramicroelectrodes, focusing on charge transfer kinetics, nucleation energetics, electrochemical reversibility, and ion dynamics at the electrode–electrolyte interface. For the first time, we demonstrate how kinetic reversibility defined by the width of the steady state kinetic regime can be used alongside the conventionally employed exchange current density (*j*_0_) to offer a more comprehensive descriptor of anode stability. Complementary density functional theory calculations provide insights into the binding energies and solvation structures of hydrated Zn^2+^ ion additive complexes revealing how additives modulate zinc ion interactions and surface reconstruction. Additive performance and stability are validated through galvanostatic cycling in symmetric Zn|Zn and asymmetric Zn|Cu cells along with Aurbach plating and stripping tests. Morphological and crystallographic analyses using SEM, XRD, and X-ray CT show distinct structural effects driven by additive chemistry. Together these electrochemical, theoretical, and structural studies establish a mechanistic framework linking molecular design to anode stabilization. Our results reveal that the remarkable performance enhancement observed with trace additives in concentrated zinc electrolytes stems from interfacial stabilization that induces kinetic irreversibility. In contrast to thermodynamic irreversibility, which is governed by energetic feasibility and is inherently a one-way process, kinetic irreversibility arises from limitations in charge transfer kinetics and the resulting overpotentials. It should not be confused with thermodynamic reversibility; two reactions may be thermodynamically reversible, yet differ in their kinetic reversibility depending on their exchange current densities and charge transfer rates, which dictate the overpotentials required to drive the reactions. A reaction with more facile charge transfer kinetics is considered more kinetically reversible than one with sluggish charge transfer, as smaller overpotentials are required to drive it in either direction. This kinetic irreversibility, alongside modified charge transfer kinetics, governs the overall electrode behavior regardless of the relative contributions from solvation or adsorption processes. While previous studies have primarily used exchange current density as the sole descriptor of charge transfer kinetics, in this work we illustrate how a complementary descriptor, the kinetic reversibility or the width of the kinetic regime can more accurately capture trends in exchange current behavior. The principles and methodologies demonstrated here are broadly applicable to other battery chemistries and the development of electrolyte evaluation protocols for comprehending anode stability.

## Results and discussion

Although the designed additives have similar functional groups, the increasing number of methyl substituents lead to significant variations in the overall kinetics of electrodeposition and the stability of the zinc anode. In the following discussion we will consider three additives: HMPA, TMPA, and PA in 1 M Zn(OTf)_2_ electrolyte. These Zn(OTf)_2_-based electrolytes were investigated, each containing a different amide additive: 0.3 M HMPA, 0.3 M TMPA, and 0.012 M PA. The concentrations were selected based on the maximum solubility of each additive in the electrolyte. For PA, concentrations above 0.012 M led to chelation with Zn^2+^ ion, resulting in precipitation. TMPA would get increasingly cloudy and excess TMPA would not dissolve when the solubility limit (0.3 M) was reached. The same solubility limit was also used for HMPA to maintain consistency across samples. The concentrations discussed above were employed throughout the study. This section is divided into several subsections, starting with first-principle DFT studies, supported by precise kinetic measurements using UMEs, followed by long-term cycling stability analysis, morphological and side product analysis. This approach provides a comprehensive understanding of the additive-induced effects on the electrochemical behavior of zinc metal anodes.

### DFT studies and kinetic analysis

**Fig. 2:**
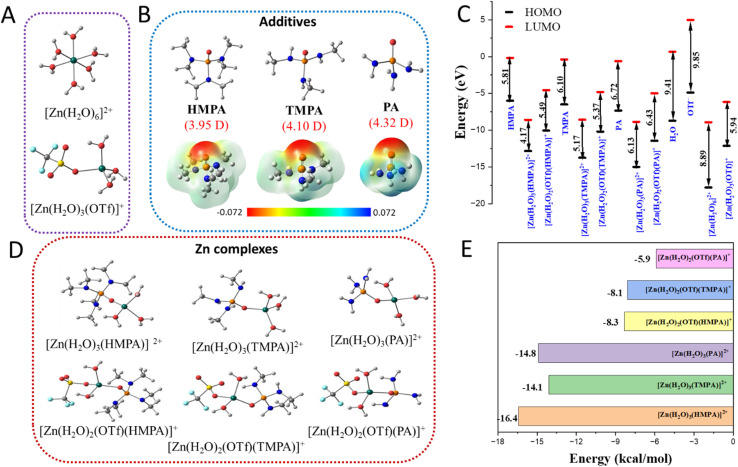
Optimized structures of the species in aqueous solution of (A) hydrated Zn ions, [Zn(H_2_O)_6_]^2+^ and [Zn(H_2_O)_3_(OTf)]^+^ (B) additives HMPA, TMPA and PA. The dipole-moments of the molecules are given in brackets. The electrostatic potential (ESP) plots of the same are shown in the panel below. (C) Relative energies of HOMO and LUMO of additive molecules and their Zn-complexes considering water and anionic ligands. (D) Optimized structures of Zn-additive complexes [Zn(H_2_O)_3_(HMPA)]^2+^, [Zn(H_2_O)_3_(TMPA)]^2+^and [Zn(H_2_O)_3_(PA)]^2+^ (top panel) Zn-additive complexes [Zn(H_2_O)_2_(OTf)(HMPA)]^+^, [Zn(H_2_O)_2_(OTf)(TMPA)]^+^ and [Zn(H_2_O)_2_(OTf)(PA)]^+^ (lower panel), where triflate anion is already bound to the Zn ion, and (E) binding energies of additive to the Zn complex. Color code: grey balls: C, white small balls: H, red balls: O, dark blue balls: N, orange balls: P, yellow balls: S, cyan balls: F, greyish green balls: Zn.

In additive-free aqueous zinc electrolytes, Zn^2+^ ions are typically octahedrally coordinated by water molecules, while in concentrated salt solutions it may also be coordinated by the anionic moiety (Fig. 2A). Upon introduction of trace amounts of organic additives such as HMPA ([(CH_3_)_2_N]_3_PO), TMPA ([(CH_3_)HN]_3_PO), and PA ([H_2_N]_3_PO) (Fig. 2B), the hydrated Zn^2+^ ions may undergo ligand exchange reactions, where one or more water molecules in the primary solvation shell are replaced by additive molecules (Fig. 2D). The preferential ligand exchange behavior is strongly dependent on the donor chemistry and steric properties of each additive. All three additives used in this study are polar molecules with varying numbers of methyl groups, resulting in the differences of their electronegativities (Fig. 2B and Table S1, SI). Among them, HMPA exhibits the lowest dipole moment and electronegativity, while PA displays the highest. Electrostatic potential (ESP) maps of the additives (Fig. 2B) reveal that the O atom in the P=O bond carries significant electron density, suggesting a favorable site for interaction with solvated Zn^2+^ ions (and the electrode, *vide infra*). The analysis of relative energies of the frontier molecular orbitals (Fig. 2C) shows that HMPA possesses a smaller energy gap between highest occupied molecular orbital and lowest unoccupied molecular orbital (HOMO–LUMO) compared to TMPA and PA, primarily due to a higher-lying HOMO. Upon complex formation with Zn^2+^, both HOMO and LUMO levels are stabilized in all three additives, leading to a significant reduction in the HOMO–LUMO gap (Fig. 2C). These findings underscore the ability of the additive molecules in modulating Zn^2+^ solvation and, consequently, influence solvation/desolvation kinetics. The relative affinity and potency of the different additives in modulating the solvation matrix are discussed in the following section.

The relative affinities of different additives towards Zn^2+^ ions depend on several key factors. Firstly, a comparison of the molecular sizes reveals that HMPA is the largest among the three additives, which may lead to slower diffusion kinetics (Fig. S3A and B). To further understand their interaction tendencies, the Gibbs free energy of solvation was calculated for the free additive molecules and for [Zn(H_2_O)_6_]^2+^ in aqueous medium. The results indicate that all additive molecules possess significantly lower solvation free energies compared to hydrated Zn^2+^ ion [Zn(H_2_O)_6_]^2+^ (Fig. S3C). Notably, HMPA exhibits the lowest solvation energy (−6.9 kcal mol^−1^), suggesting that it undergoes desolvation more readily than TMPA or PA. This lower solvation energy facilitates ligand exchange with hydrated Zn^2+^ ions, thereby enabling modulation of the solvation environment.

The Gibbs free energies (in water, Δ*G*_w_) calculated for the ligand exchange reactions between additive molecules and octahedrally solvated Zn^2+^ ions are presented below. The values indicate that the formation of the tetrahedral Zn-additive complex is spontaneous for all additives. The reactions are as follows:[Zn(H_2_O)_6_]^2+^ + HMPA → [Zn(H_2_O)_3_(HMPA)]^2+^ + 3H_2_O, Δ*G*_w_ = −16.4 kcal mol^−1^[Zn(H_2_O)_6_]^2+^ + TMPA → [Zn(H_2_O)_3_(TMPA)]^2+^ + 3H_2_O, Δ*G*_w_ = −14.1 kcal mol^−1^[Zn(H_2_O)_6_]^2+^ + PA → [Zn(H_2_O)_3_(PA)]^2+^ + 3H_2_O, Δ*G*_w_ = −14.8 kcal mol^−1^

The optimized structures of the Zn-complexes are shown in Fig. 2D. The free energies of the ligand exchange reactions are found to be similar, with HMPA binding being slightly more favorable compared to TMPA and PA (Fig. 2E). Additionally, structural parameters such as bond distances (O–P: 1.53 Å, Zn–O_P_: 2.03 Å, Zn–OH: 2.06 Å) and angles for all the Zn-complexes were found to be consistent in the presence of these additives. Since only trace amounts of additives are typically added to the electrolytic solution (see SI for details), we can rule out the possibility of replacing more than one water molecule in the hydrated [Zn(H_2_O)_6_]^2+^ with ligand molecules.

Given the use of a highly concentrated electrolyte (1 M), it is essential to consider the presence of the triflate counterion (OTf^−^) in solution, which originates from the dissociation of the Zn salt and may interact with Zn^2+^ ions in the aqueous phase. Our analysis indicates that OTf^−^ can coordinate to Zn^2+^ either in a monodentate or bidentate fashion (see Table S2). The resulting Zn-OTf complex adopt a tetrahedral geometry (Fig. 2B, lower panel), in contrast to the octahedral geometry of [Zn(H_2_O)_6_]^2+^. Accordingly, we considered the following ligand exchange reactions between additive molecules and Zn^2+^ ions already coordinated to OTf^−^ in a monodentate mode in aqueous medium.[Zn(OTf)(H_2_O)_3_]^+^ + HMPA → [Zn(OTf)(H_2_O)_2_(HMPA)]^+^ + H_2_O, Δ*G*_w_ = −8.3 kcal mol^−1^[Zn(OTf)(H_2_O)_3_]^+^ + TMPA → [Zn(OTf)(H_2_O)_2_(TMPA)]^+^ + H_2_O, Δ*G*_w_ = −8.1 kcal mol^−1^[Zn(OTf)(H_2_O)_3_]^+^ + PA → [Zn(OTf)(H_2_O)_2_(PA)]^+^ + H_2_O, Δ*G*_w_ = −5.9 kcal mol^−1^

From the results presented in Fig. 2E and Table S3, it is evident that HMPA binds more strongly to Zn^2+^ ions compared to TMPA and PA in an aqueous medium at realistic additive concentrations, where the influence of the counter anion cannot be neglected. The corresponding Zn–OTf complexes are also shown in Fig. 2D, bottom panel. The preferential solvation ability of the additive molecules follows the order: HMPA > PA ≈ TMPA, with PA and TMPA exhibiting similar behavior. This trend provides insight into the differences observed in charge transfer kinetics using UMEs, where the addition of HMPA results in the lowest *j*_0_ value, followed by TMPA and PA, respectively. In essence, a lower *j*_0_ value is closely associated with a stronger solvation matrix, which imposes a higher charge transfer resistance due to the increased energy barrier for desolvation prior to zinc electrodeposition. Therefore, the theoretical findings based on ligand exchange affinities clearly explain the observed trend in *j*_0_ values, with HMPA exhibiting the strongest binding and the lowest charge transfer rate, followed by PA and TMPA. Another important factor that may influence the kinetics of zinc electrodeposition in the presence of additives is the preferential binding of trace additive molecules to the surface of the zinc anode. To investigate this aspect, we considered the electrodeposition process leading to the nucleation of small metallic zinc clusters. Specifically, we modeled a neutral Zn_4_ cluster and evaluated the binding free energies of additive molecules on this cluster (see structures in Fig. S4A–C). In addition, we examined the binding free energies of water, the triflate anion (OTf^−^: CF_3_SO_2_O^−^), and hydroxyl ion (OH^−^) on the Zn_4_ cluster, as these ionic species are prevalent in the electrolytic medium (Table S4, Fig. S4D–F). The respective binding energies were found to be −2.6 kcal mol^−1^ for water and −21.1 kcal mol^−1^ for triflate anion, while the hydroxyl ion exhibited significantly stronger binding with an energy of −71.2 kcal mol^−1^. We further calculated the binding energies of HMPA, TMPA, and PA on the Zn_4_ cluster. The results show that HMPA binds with a free energy of −14.5 kcal mol^−1^, followed by TMPA (−13.8 kcal mol^−1^) and PA (−12.2 kcal mol^−1^). In these cases, the Zn–O_P_ and O–P bond distances increase to 2.09 Å and 1.52 Å, respectively, which are longer than those observed for the ionic species, indicating weaker interaction between the metallic Zn_4_ cluster and the additives. Notably, the weaker binding of PA (−12.2 kcal mol^−1^) is reflected in a further elongation of the Zn–O bond to 2.14 Å. These results demonstrate that despite all three additives possessing the same coordinating center (P=O) capable of interacting with zinc ions and altering the solvation environment, there are pronounced differences in their overall lability due to the surrounding chemical moieties—specifically, hydrogen *versus* methyl groups. The presence of six methyl groups in HMPA leads to the lowest dipole moment and the smallest HOMO–LUMO energy gap among the additives, compared to TMPA (three methyl groups) and PA (no methyl groups). These electronic differences significantly influence the additives' ability to modulate the solvation matrix and, consequently, influence the electron transfer kinetics during zinc electrodeposition.

**Fig. 3:**
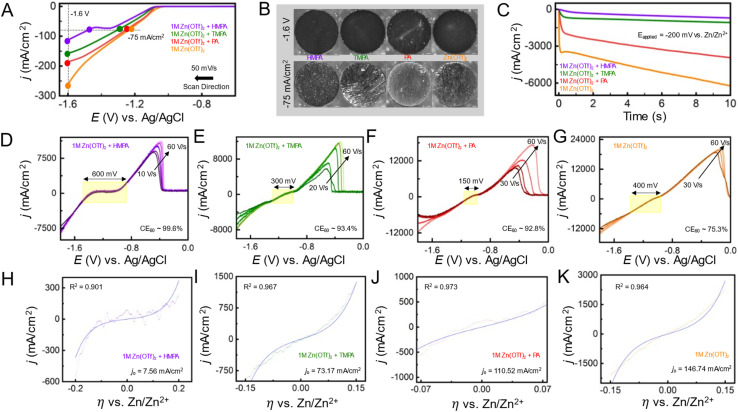
(A) Linear sweep voltammogram on the Cu macroelectrode in different electrolytes containing additives HMPA, TMPA, PA in 1 M Zn(OTf)_2_. (B) *In situ* optical microscopy snapshots at −1.6 V and at −75 mA cm^−2^ during the voltammetric sweep for different electrolytes. (C) Chronoamperometry to study electrodeposition of the Zn(OTf)_2_ electrolytes at an overpotential step of −200 mV *vs.* Zn/Zn^2+^. (D–G) Backward sweep of the fast scan cyclic voltammograms with kinetically controlled low-overpotential regimes as a function of scan rates (10–60 V s^−1^ for HMPA, 20–60 for TMPA, 30–60 for PA and bare Zn(OTf)_2_ electrolyte), showing the width of kinetic regime as well as the coulombic efficiency for the voltammograms at 60 V s^−1^ (H–K).

We utilize fundamental electrochemical measurements on macroelectrodes, coupled with optical microscopy and UMEs, to validate findings from first-principles calculations on how modulation of the solvation matrix in presence of additives (HMPA, PA, and TMPA) influences electron transfer kinetics during zinc electrodeposition. Firstly, linear sweep voltammetry (LSV) was performed using a three-electrode setup consisting of a 1 mm copper disk as the working electrode, a zinc counter electrode, and an Ag/AgCl reference electrode (in 1 M KCl). The scans were conducted from −0.6 V to −1.6 V *vs.* Ag/AgCl at a scan rate of 50 mV s^−1^, as shown in the scan direction in Fig. 3A. All measurements were carried out in a Teflon electrochemical cell with the copper disk vertically mounted and monitored *in situ* using a high-resolution camera positioned above the cell, enabling real-time visualization of the electrode surface. The pH of the Zn(OTf)_2_ based electrolytes were measured and the pH of the bare electrolyte was measured to be 5.47. Upon the addition of HMPA, the pH decreased to 4.35, while TMPA and PA resulted in pH values of 5.28 and 4.79. The pH is important, as lower pH environments can influence zinc metal anode stability by promoting side reactions such as hydrogen evolution and corrosion.^49^ Therefore, understanding how each additive affects the pH helps to elucidate their role in stabilizing the zinc interface and improving overall battery performance. As the potential was swept negatively, an increase in current density was observed, attributed to both increasing electroactive surface area and mass transport limitations. A lower rise in current density is indicative of more uniform deposition and potentially slower electron transfer kinetics. Fig. 3A compares the voltammograms of the additive-free Zn(OTf)_2_ electrolyte with those containing each additive. Among them, the HMPA-containing system exhibited the lowest current density at −1.6 V *vs.* Ag/AgCl, while the additive-free Zn(OTf)_2_ system showed the highest. The observed trend in current density at −1.6 V was: HMPA < TMPA < PA < Zn(OTf)_2_. Based on this, we speculate that the kinetics of zinc electrodeposition follow the opposite trend of current density, with HMPA exhibiting the most sluggish kinetics. Corresponding *in situ* optical micrographs at −1.6 V and a current density of −75 mA cm^−2^ are shown in Fig. 3B, revealing marked differences in deposition morphology. In the absence of additives, Zn(OTf)_2_ exhibited pronounced edge effects and dendritic growth along the periphery of the copper disk. A zoomed in image of the periphery copper disk can be shown in Fig. S5. In contrast, the HMPA-containing electrolyte yielded smoother and more uniform deposition, with significant suppression of dendritic features. These distinct growth patterns, observed at a fixed current density, correlate well with the reduction behavior and interfacial kinetics of each electrolyte system.

To gain deeper insights into the nucleation and growth energetics of Zn electroplating, chronoamperometry (CA) was employed to probe the nucleation overpotential. The CA experiments were conducted using a 12.5 µm tungsten microelectrode as the working electrode and a zinc foil serving as both the counter and reference electrode. A potential step was applied from an initial value of +200 mV to −200 mV *vs.* Zn/Zn^2+^, with a pulse width of 10 seconds at the reductive potential. The chronoamperometric data for each electrolyte system is presented in Fig. 3C. The Zn(OTf)_2_ electrolyte exhibited the highest initial current density peak and a pronounced increase in current density over the 10 second pulse, indicative of rapid, non-uniform, and dendritic Zn deposition. In contrast, the electrolyte containing HMPA showed the lowest initial current density and the smallest change over time, suggesting more uniform and controlled nucleation and growth. TMPA followed a similar trend to HMPA but exhibited a slightly higher initial current. The PA-containing electrolyte showed a larger current peak than both HMPA and TMPA, displaying behavior closer to the additive-free Zn(OTf)_2_ baseline. Taken together with the LSV results in Fig. 3A, it is evident that HMPA leads to the most sluggish electron transfer kinetics, which correlates with the most uniform zinc morphology, followed by TMPA, PA, and the bare 1 M Zn(OTf)_2_ electrolyte. The kinetic aspects of electrodeposition in the presence of these additives are explored in the following section using fast-scan voltammetry (FSCV).

FSCV has previously been employed by our group to accurately probe the kinetics of zinc electrodeposition.^50–52^ We demonstrated that by rapidly sweeping the potential, it is possible to isolate the kinetics of zinc electrodeposition near the equilibrium potential. This approach minimizes the influence of mass transfer limitations, which can often obscure kinetic measurements at slower scan rates, leading to inaccurate interpretations. For instance, Fig. 3D illustrates the forward sweep for FSCV recorded at scan rates ranging from 10 V s^−1^ to 60 V s^−1^, where the kinetic regime, indicated within the yellow box, remains independent of the scan rate. At scan rates lower than 20 V s^−1^, the kinetic regime is convoluted by mass transfer effects, as shown in Fig. S6. The scan rate at which mass transfer limitations become negligible is referred to as the “cutoff” scan rate, which delineates the boundary for the “fast scan” regime. The cutoff scan rate has been established as a reliable metric for understanding the balance between kinetics and mass transfer, where a lower cutoff signifies sluggish charge transfer kinetics, as demonstrated in our previously reported work.^51,52^ Ideally, we aim to isolate scan rates where the kinetic regime becomes independent of the scan rate of the voltammetric sweep. Similarly, Fig. 3E–G display the backward sweep of voltammograms obtained at various scan rates within the “fast scan” region for each electrolyte system. Based on the data presented in Fig. S7, the cutoff scan rates were observed to be 10 V s^−1^ for HMPA, 20 V s^−1^ for TMPA, and 30 V s^−1^ for PA and bare electrolyte. This suggests that HMPA exhibits the most sluggish electron transfer kinetics, as a lower cutoff scan rate causes the kinetic regime to transition into one largely unaffected by mass transfer effects, followed by TMPA, PA, and bare electrolyte. The FSCV data also enables a comparison of the coulombic efficiencies (CE) across this electrolyte system by converting the applied voltage ramp as a function of time. We performed the comparison at a scan rate of 60 V s^−1^. The CE values at 60 V s^−1^, based on the data shown in Fig. S8 and 3D–G, are 99.93%, 93.4%, 92.8%, and 75.3% for HMPA, TMPA, PA, and bare electrolyte, respectively. Both the cutoff scan rates and CE values demonstrate that HMPA exhibits the most sluggish charge transfer kinetics along with the highest reversibility (*i.e.*, CE), followed by TMPA, PA, and bare electrolyte. These trends align with the findings from optical microscopy and CA experiments presented earlier.

To further investigate the charge transfer kinetics, we performed additional analysis of the kinetic regimes by fitting them with kinetic models of charge transfer, as well as assessing the width (voltage window) that explains the extent of kinetic irreversibility in the presence of additives. Firstly, the width of the kinetic regime, highlighted by the yellow shaded regions in Fig. 3D–G, was found to be approximately 600 mV for HMPA, 300 mV for TMPA, 150 mV for PA, and 400 mV for the bare electrolyte. As expected, HMPA exhibits the widest kinetic regime, indicative of sluggish charge transfer kinetics and a greater degree of kinetic irreversibility compared to TMPA, PA, and the bare electrolyte. Concentration-dependent effects were studied, specifically for PA that would precipitate at higher levels. In Fig. S9, different concentrations of PA were tested. At the highest concentration of 12 mM the kinetic width was 150 mM with a *j*_0_ of 110.52 mA cm^−2^ while the lowest concentration of PA (1 mM) had a kinetic width of 50 mV and a *j*_0_ of 197.38 mA cm^−2^. As the concentration increases, the kinetic width increases and the *j*_0_ decreases. This broader voltage window reflects the increased energy barrier for charge transfer in the presence of HMPA, further supporting its characterization as the most kinetically irreversible system among those studied. It is important to note that kinetic irreversibility should not be confused with thermodynamic irreversibility. The former refers specifically to the sluggishness of charge transfer arising from kinetic limitations rather than thermodynamic driving force. In this context, a broader kinetic regime reflects a slower charge transfer process, even when the overall redox reaction remains thermodynamically reversible.

To accurately quantify the charge transfer kinetics, the kinetic regime was analyzed using the Butler–Volmer (BV) formulation through nonlinear least-squares fitting, with the exchange current density (*j*_0_) as the sole fitting parameter. This approach allowed for consistent and comparative evaluation of the electron transfer kinetics across the different electrolytes. This method, previously detailed in our earlier work, enables a direct assessment of the intrinsic rate of electron transfer at equilibrium, providing a robust measure of charge transfer kinetics. For each electrolyte system, the FSCV data were fitted to the BV model, which describes the current–overpotential equation (*i vs. η*--see SI for details). The kinetic regimes (dotted lines) for all electrolytes, along with their respective fits using the BV model (solid lines), are shown in blue in Fig. 3H–K. A detailed comparison reveals that the HMPA-containing electrolyte exhibited the lowest exchange current density, with a calculated *j*_0_ of 7.56 mA cm^−2^. This result aligns well with earlier observations, including the broadest kinetic regime and the highest CE, collectively indicating significantly suppressed charge transfer kinetics, an effect consistent with more uniform and controlled Zn electrodeposition. In comparison, the TMPA-containing electrolyte (Fig. 3I) yielded a *j*_0_ of 73.17 mA cm^−2^, suggesting moderately enhanced charge transfer kinetics relative to HMPA. The highest *j*_0_ values were observed for the PA-containing electrolyte and the bare Zn(OTf)_2_ electrolyte, as shown in Fig. 3J and K, with values of 110.52 mA cm^−2^ and 146.74 mA cm^−2^, respectively. These findings establish a clear trend: the incorporation of HMPA most effectively slows Zn plating kinetics, promoting more uniform deposition accompanied by a kinetically irreversible regime, whereas the baseline Zn(OTf)_2_ system exhibits the fastest, and consequently less controlled, electrodeposition behavior. TMPA and PA represent intermediate cases, with progressively faster kinetics and narrower kinetic regimes. The overall findings presented in Fig. 3 are summarized in Table 1, providing a comparative overview of key electrochemical parameters, such as cutoff scan rates, kinetic regime widths, exchange current densities (*j*_0_), and coulombic efficiencies, for each electrolyte system. This summary underscores the distinct influence of electrolyte composition on zinc electrodeposition kinetics and reversibility. The higher binding energy of HMPA, followed by TMPA and PA, toward metallic Zn^0^ clusters, along with their ability to modulate solvation energetics in the order HMPA > PA > TMPA, collectively explain the observed kinetic characteristics of zinc electrodeposition in the presence of these electrolyte additives. It is important to note that both of these factors, the strong substrate binding affinity and the significant alteration of solvation structure, are most pronounced for HMPA. This dual influence likely contributes to the irreversible kinetic behavior observed in Fig. 3, marked by the lowest *j*_0_ values and, consequently, the highest CE measured *via* voltammetry.

**Table 1:** Comparison of kinetic parameters for HMPA, TMPA, PA, and bare 1 M Zn(OTf)_2_ electrolytes. The table summarizes key electrochemical metrics including the cutoff scan rate (defining the transition to the fast scan regime), the width of the kinetic regime (reflecting kinetic irreversibility), coulombic efficiency (CE) at 60 V s^−1^, and the exchange current density (*j*_0_) obtained from Butler–Volmer fitting

Electrolyte	Cutoff scan rate (V s^−1^)	Kinetic regime width (mV)	CE @ 60 V s^−1^ (%)	*j*_0_ (mA cm^−2^)
HMPA	10	600	99.6	7.56
TMPA	20	300	93.4	73.17
PA	30	150	92.8	110.52
Bare Zn(OTf)_2_	30	400	75.3	146.74

The results, together with the stability data (*vide infra*) and charge transfer kinetics reflected by exchange current density values, clearly demonstrate that kinetic reversibility is a powerful and complementary descriptor for assessing anode stability, extending beyond the conventional use of exchange current density alone. We encourage its broader adoption within the research community as a meaningful tool to evaluate and design more robust zinc metal anodes. Overall, the combination of theoretical modeling and kinetic analysis presented in this study highlights the importance of a mechanistic understanding of additive chemistry that goes beyond standard electrochemical characterization. By uncovering the molecular-level roles of electrolyte additives through insights into surface binding affinities and solvation energetics, we establish a more complete picture of additive–electrode interactions. We now proceed to examine coin cell performance and its correlation with the kinetic properties presented in Table 1.

### Long-term stability of anode in AZMBs

**Fig. 4:**
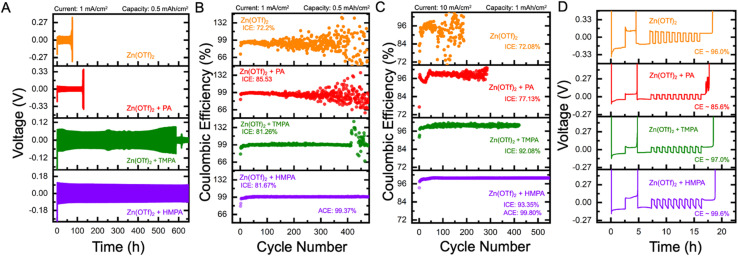
(A) Symmetric cell cycling of different electrolytes containing additives HMPA, TMPA, PA in 1 M Zn(OTf)_2_ at a current density of 1 mA cm^−2^ and capacity of 0.5 mA h cm^−2^. (B) Asymmetric cell cycling of different electrolytes containing additives HMPA, TMPA, PA in 1 M Zn(OTf)_2_ at a current density of 1 mA cm^−2^ and capacity of 0.5 mA h cm^−2^ with initial coulombic efficiency (ICE) for all and average coulombic efficiency (ACE) for electrolyte containing HMPA. (C) High rate asymmetric cell cycling of different electrolytes containing additives HMPA, TMPA, PA in 1 M Zn(OTf)_2_ at a current density of 10 mA cm^−2^ and capacity of 1 mA h cm^−2^ with initial coulombic efficiency (ICE) for all and average coulombic efficiency (ACE) for electrolyte containing HMPA. (D) Representative potential *vs.* time curve of different electrolytes containing additives HMPA, TMPA, PA in 1 M Zn(OTf)_2_ using a galvanostatic charge–discharge protocol. In this protocol, an initial conditioning cycle was conducted to minimize substrate effects. During the conditioning cycle, 5 mA h cm^−2^ of Zn was plated onto the Cu electrode. The Zn on the Cu electrode was then stripped at a current density of 2 mA cm^−2^ with a cutoff voltage of +0.5 V. After the conditioning cycle, a limited and controlled source of Zn was plated for precise CE determination. Next, the cell was cycled 9 times at a current density of 2 mA cm^−2^ and a capacity of 1 mA h cm^−2^ with a cut off voltage of +0.5 V *versus* Zn/Zn^2+^, ensuring all the plated Zn was stripped.

Based on the discussion thus far, it is evident that the electrolyte containing HMPA facilitates uniform and non-dendritic Zn deposition and exhibits the lowest *j*_0_ value compared to TMPA and PA. More importantly, a similar trend was observed in the CE values measured using FSCV. To evaluate the long-term stability of the Zn anode, coin cell cycling studies were conducted under both Zn‖Zn symmetric and Cu‖Zn asymmetric configurations. As shown in Fig. 4A, the symmetric cells were galvanostatically cycled at a current density of 1 mA cm^−2^ with a capacity of 0.5 mA h cm^−2^. As expected, the electrolyte containing HMPA exhibited the highest Zn anode stability, enabling stable cycling for over 700 cycles, significantly outperforming the other additive-containing electrolytes. The TMPA-based electrolyte provided moderate stability, with a short circuit occurring just before the 600th cycle. In contrast, the PA-containing electrolyte showed poor anode stability, with short-circuit failure observed before the 150th cycle. The additive-free baseline electrolyte containing only Zn(OTf)_2_ demonstrated the least stability, short-circuiting before 100 cycles. This trend is consistent with the findings presented earlier. Additionally, the nucleation overpotentials (*η*_nuc_) for all electrolytes, which directly correlate with the *j*_0_ from the kinetic analysis are shown in Fig. S10. As expected, the HMPA-containing electrolyte exhibits the highest *η*_nuc_, reflecting sluggish electrodeposition kinetics due to the strong solvation and surface-binding effects of HMPA. In contrast, the additive-free Zn(OTf)_2_ electrolyte displays the lowest *η*_nuc_, consistent with the absence of any kinetic hindrance from additives, as discussed in Fig. 3D and G. The TMPA and PA additives show intermediate *η*_nuc_ values, aligning with their respective *j*_0_ values. The observed trend in nucleation overpotentials follows the order: HMPA > TMPA > PA > Zn(OTf)_2_, which is in agreement with the trend in *j*_0_ values (lowest to highest: HMPA < TMPA < PA < Zn(OTf)_2_).

Under asymmetric Cu‖Zn coin cell cycling tests, cells were galvanostatically charged and discharged at a current density of 1 mA cm^−2^ with a capacity of 0.5 mA h cm^−2^ (cutoff voltage: 0.6 V *vs.* Zn/Zn^2+^).^53^ As shown in Fig. 4B, the electrolyte containing HMPA demonstrated the best overall performance, with an initial coulombic efficiency (ICE) of 81.67% and exceptional long-term stability, maintaining consistent cycling beyond 400 cycles and achieving an average coulombic efficiency (ACE) of 99.37%. The TMPA-containing electrolyte exhibited a slightly lower ICE of 81.26%, with CE remaining stable until around the 400th cycle, after which a noticeable decline was observed. The PA-containing electrolyte, while showing the highest ICE at 85.53%, suffered from poor long-term reversibility, with CE fluctuations emerging around 150 cycles. A similar trend was observed for the additive-free Zn(OTf)_2_ electrolyte, which started with a lower ICE of 72.20% and displayed severe CE instability beyond 150 cycles. High-rate cycling (10 mA cm^−2^ 0.5 mA h cm^−2^) was performed as shown in Fig. 4C to show the robustness of the additives. The electrolytes containing HMPA showed the best overall performance with an ICE of 93.35%. The TMPA containing electrolyte exhibited a lower ICE of 92.06%. It short-circuited a little past the 400th cycle while HMPA is still cycling. The PA containing electrolyte showed an ICE of 77.13% which short-circuited around the 300th cycle. The additive-free Zn(OTf)_2_ electrolyte did not perform well compared to the additive containing electrolytes with an ICE of 72.08%. Variations in CE measurements across coin cells can be strongly impacted by substrate-related factors, including lattice mismatch, alloy formation, and interfacial dynamics. To minimize these effects and enable meaningful comparison between different electrolytes, a galvanostatic protocol proposed by Adams *et al.* and Xu *et al.*^54,55^ was employed, as shown in Fig. 4D (refer to the figure caption for details). This protocol is widely regarded as a more accurate method for determining CE in asymmetric coin cell formats. Using this approach, the CE was found to be poor for both the PA additive (85.6%) and bare Zn(OTf)_2_ (96.0%). The TMPA additive showed moderate performance with 97.0% CE, while the HMPA additive exhibited excellent CE of 99.6%.

Overall, the coin cell cycling experiments clearly demonstrate that across symmetric, asymmetric, and galvanostatic charge–discharge protocols, the HMPA-containing electrolyte consistently delivers the highest stability, while the bare electrolyte shows the poorest performance. TMPA and PA exhibit intermediate behavior. These results strongly correlate with the kinetic parameters extracted from fast scan cyclic voltammetry, particularly the exchange current density and kinetic reversibility, and are further supported by theoretical modeling of solvation and interfacial interactions. The ability of kinetic analysis to differentiate between additive chemistries provides mechanistic insight into the underlying causes of performance disparities in full-cell configurations. In particular, kinetic reversibility captures features of interfacial stability and charge transfer efficiency that are not evident from traditional descriptors alone. This connection between kinetic properties and practical cycling behavior highlights the utility of UME-based electroanalysis in guiding rational additive design. By linking molecular-level kinetics to macroscopic battery performance, this approach establishes a foundation for the predictive development of new additives aimed at enhancing the durability and reversibility of zinc metal anodes.

### Morphological analysis and competitive surface adsorption kinetics

**Fig. 5:**
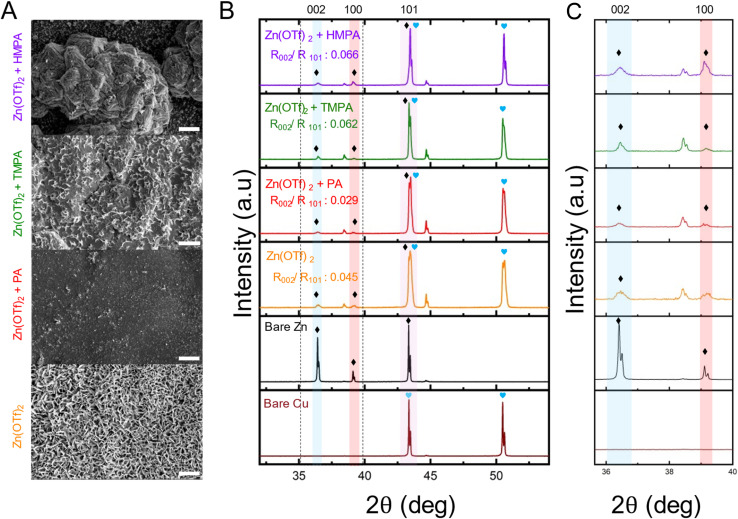
Post cycling characterizations: (A) SEM images showing morphology of electrolytes containing additives HMPA, TMPA, PA in 1 M Zn(OTf)_2_ from galvanostatic deposition of zinc on Cu in a Cu|Zn asymmetric cell. (B) XRD spectra of the electrodeposited samples of electrolytes containing HMPA, TMPA, PA in 1 M Zn(OTf)_2_ along with bare Zn and Cu. (C) XRD spectra of the electrodeposited samples from 35–40°. The scale bar for (A) is 1 µm.

Enhanced zinc metal anode stability and performance critically depend on achieving uniform and compact metal deposition. Such morphology not only preserves structural integrity during repeated plating and stripping cycles but also mitigates dendrite formation and suppresses parasitic side reactions, thereby improving long-term cycling efficiency and overall battery lifespan. Fig. 5A shows the zinc morphology after galvanostatic deposition on Cu in Cu|Zn asymmetric cells at a current of 5 mA cm^−2^ and a capacity of 2.5 mA h cm^−2^ using different electrolytes. With the HMPA additive, zinc deposition is notably more uniform and compact which are key characteristics that contribute to improved anode stability in AZMBs. Uniform deposition ensures even current distribution across the anode surface, minimizing localized hotspots and dendrite formation. Compact growth further reduces the likelihood of loosely bound zinc and the formation of inactive “dead” zinc. In contrast, the TMPA additive promotes more uniform growth compared to the control but lacks compactness. The PA additive results in inadequate and uneven deposition, consistent with the lower CE values observed in coin cell tests. The bare Zn(OTf)_2_ electrolyte shows the poorest performance, with highly non-uniform, flake-like zinc platelets. This morphology is attributed to the formation of insulating zinc hydroxide hydrate byproducts, which disrupt Zn^2+^ ion transport and lead to heterogeneous nucleation. Such non-uniform growth exacerbates current localization, promotes dendrite formation, and ultimately compromises both the structural and electrochemical stability of the zinc anode in AZMBs.

It is well established in literature that compact zinc deposition is often associated with preferential growth along the (002) crystallographic plane.^56–58^ To investigate this, X-ray diffraction (XRD) analysis was performed to characterize the orientation of electrodeposited zinc in the presence of HMPA, TMPA, and PA additives in 1 M Zn(OTf)_2_ (Fig. 5B). Distinct diffraction peaks corresponding to the low surface energy (002) facet, along with the higher energy (100) and (101) planes, confirm the formation of hexagonal close-packed (hcp) zinc and its preferred growth orientations. A higher relative intensity of the (002) peak is particularly favorable, as it correlates with compact and uniform deposition—morphological features that are critical for suppressing dendrite formation, reducing surface roughness, and enhancing the electrochemical stability and reversibility of the zinc anode. Among the tested electrolytes, the HMPA additive resulted in the highest (002)/(101) intensity ratio, indicating strong preferential orientation toward the basal (002) plane and thus the most favorable deposition behavior. TMPA exhibited the second-highest ratio, followed by the bare Zn(OTf)_2_ electrolyte, while the PA additive showed the lowest (002)/(101) ratio. The additional peaks observed in the XRD patterns, aside from the indexed zinc orientations, originate from the underlying Cu foil substrate. Additionally, XRD analysis was conducted on bare Cu and Zn foil substrates to establish reference diffraction peaks for each metal. These baseline peaks were used to identify and assign characteristic peaks in electrodeposited or composite samples. Distinct diffraction peaks corresponding to Cu (blue heart) and Zn (black diamond) were clearly marked to enable accurate differentiation. However, it is important to note that around 43°, the Cu (111) and Zn (101) peaks overlap, which can complicate phase identification. To ensure accurate interpretation, careful comparison with reference patterns is required to distinguish the Zn (101) peak from the overlapping Cu (111) signal. The overlap of Zn (101) with Cu (111) peaks indicates similar interplanar spacings, which may promote preferential crystallographic alignment. This alignment may lower the interfacial energy at the Zn–Cu interface, potentially favoring Zn nucleation and leading to more uniform initial deposition.

Fig. 5C showcases a part of the XRD spectra (35–40°) which was taken to show the low surface energy facet (002) comparison for the electrolytes containing additive HMPA, TMPA, and PA in 1 M Zn(OTf)_2_. Although the low surface energy facet (002) peaks exist with relatively low intensity across all samples, a clear trend can still be observed. Among the tested electrolytes, the additive-containing electrolyte with HMPA exhibits the highest (002) peak intensity, followed by TMPA and the bare Zn(OTf)_2_ electrolyte without additives. In contrast, the electrolyte containing PA shows the lowest (002) peak intensity. This variation suggests that the degree of preferential growth along the (002) facet. The low surface energy orientation associated with uniform and compact Zn deposition is strongly influenced by the specific additive used. This trend in crystallographic orientation closely aligns with the surface morphologies observed *via* SEM in Fig. 5A, further reinforcing the critical role of electrolyte additives in modulating zinc deposition pathways and enhancing zinc metal anode performance.

To eliminate any ambiguities arising from localized area selection in SEM and XRD analyses, X-ray computed tomography (X-ray CT) was employed. X-ray CT is a powerful, non-destructive technique that allows three-dimensional visualization of the internal structure of zinc deposits at larger length scales than SEM. This enables comprehensive assessment of the entire electrode surface in a single frame, offering deeper insights into deposition uniformity, porosity, and potential dendritic growth. Such holistic morphological characterization is crucial for understanding how zinc deposition behavior influences the long-term stability and performance of the zinc metal anode during cycling. The X-ray CT images for all the electrolytes are presented in Fig. S11. The zoomed in images for all the cases are shown in shown in Fig. S12 which shows the zinc deposition from the electrolyte containing the HMPA, TMPA, PA additive and bare electrolyte on a Cu substrate. The deposition for all the cases can be seen for all the electrolytes, with HMPA appearing both uniform and compact, with TMPA less compact, with PA poor and uneven zinc coverage, and bare electrolyte with non-uniform and signs of uncontrolled dendritic growth. The contour plots shown in Fig. S13 reveal different levels of porosity and presence of deep voids, indicating the effects of depositions with each electrolyte. Moreover, trace additives significantly altered the electrolyte pH (4.35 for HMPA, 5.28 for TMPA, 4.79 for PA, and 5.47 for the bare electrolyte). Since electrolyte pH strongly affects HER kinetics, the initial H_2_ evolution during plating follows this trend (Fig. S14). The most acidic system, HMPA, shows the highest H_2_ evolution in the first cycle followed by marked suppression in the second; a similar trend is seen for PA, while TMPA and the bare electrolyte show minimal change. Although overall HER suppression is limited, the dynamic decrease in H_2_ evolution after the first cycle may be more critical than the absolute rate in governing anode stability.

From the morphological and surface texturing analyses presented in Fig. 5, it can be concluded that the electrolyte condition corresponding to HMPA additive is ideal compared to PA, TMPA and bare cases for enhancing zinc metal anode stability. The addition of HMPA leads to an optimal surface texture that promotes long-term stability during repeated cycling. Overall, the superior performance can be attributed to two key factors: first, the kinetic arguments discussed earlier, particularly solvation modulation; and second, the competitive surface adsorption of additives, which is examined in detail in the next section.

Two distinct techniques have been reported recently to control the dendrite formation on the electrode surface, inclusion of additives which facilitates the homogeneous Zn nucleation, and locking Zn platelets *via* electrochemical epitaxy.^59,60^ Previous reports on the role of additives on the Zn anode directs towards the importance of preferential adsorption of additives/molecules over Zn (101) rather than Zn (002) orientations.^60–62^ It was claimed that the competitive adsorption of additives over Zn (002) orientation hinders the deposition of Zn ion over Zn (101) surface, resulting in a comparatively uniform deposition and inhibition of Zn dendrite formation.^59–62^ To investigate the role of the additives on the zinc anode, we modeled both the Zn (002) and Zn (101) facets and calculated the adsorption energies of each molecule, enabling a comparative evaluation of their binding affinities to specific zinc facets. The surface energies of the two zinc facets are different, 0.068 eV (002), and 0.128 eV (101) (details in Fig. S16), which indicates that the former is the more thermodynamically stable, which is in accordance with earlier studies.^61,62^ Here, hexagonal close-packed crystallographic anisotropy governs the lower surface energy of Zn (002), also seen experimentally.^59^ We then calculated the binding energies of all the important species present (Table S5) onto Zn (002) and Zn (101) surfaces.

**Fig. 6:**
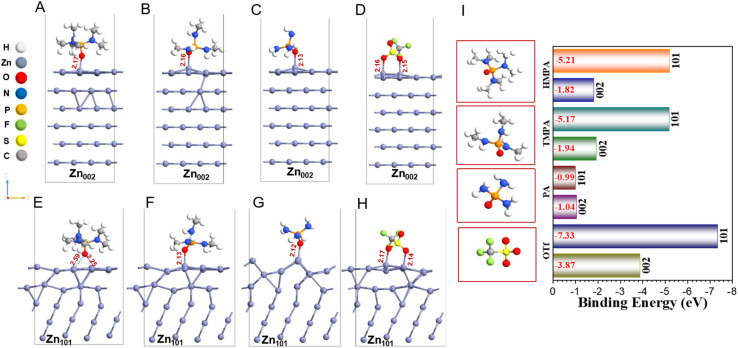
Optimized structures of HMPA, TMPA, PA, and OTf additives adsorbed over Zn (002) (A–D), and Zn (101) surface (E–H), and (I) calculated binding energies of additives on (002) and (101) zinc facets on the electrode. The binding energies are written in red. All bond lengths are in Å.

Adsorption of additives, HMPA, TMPA, and PA, on Zn (002), Zn (101) surface shows interesting interfacial chemistry. The binding of PA is weakest, −1.04 eV (002), and −0.99 eV (101) among other additive molecules. The binding in this case is “on-top” with Zn–O bond-distance of 2.13 Å (002) and 2.12 Å (101) respectively (Fig. 6). The size of the PA molecule is small (end-to end distance ∼4.2 Å), but the steric interaction of the H atoms of PA with surface Zn atoms can be clearly distinguished between Zn (002), and Zn (101) surfaces, which affects the binding energies. Larger ligands, HMPA and TMPA occupies a larger area on the Zn electrode and with more methyl groups now interacting with Zn surface, the binding energies are modified, even though the binding is still through the P=O bond. HMPA on one hand is adsorbed “on-top” over Zn (002) with Zn–O bond-distance of 2.17 Å, but on “fcc hollow” site for Zn (101) with 2.59/2.25 Å bond lengths (Fig. 6A and E). Whereas TMPA in either surface is bound “on-top” with 2.16/2.13 Å Zn–O bond length for (002)/(101) (Fig. 6B and F). From binding energies of HMPA, and TMPA, we see both additives are strongly adsorbed over Zn (101) surface, with −5.21 eV (HMPA), and −5.17 eV (TMPA), although for Zn (002) binding is comparatively much weaker: −1.82 eV (HMPA), and −1.94 eV (TMPA). The large difference in binding strengths of HMPA and TMPA on both surfaces is governed by the size of additives, where H-atoms of HMPA and TMPA strongly interact with surface Zn-atoms. The Zn–H interaction is stronger for Zn (101), compared to Zn (002), resulting in an additive induced surface reconstruction, which drives the Zn (101) surface to reconstruct into Zn (002), due to lower surface energy. We have also calculated the binding energies of OTf as it is present in the electrolyte in large excess compared to the trace amounts of additive molecules. We find that OTf binds strongly on Zn (002) with binding energies of −3.87 eV, but even stronger (−7.33 eV) on the Zn (101) electrode surface and the binding is bidentate in nature (Fig. 6D and H). Compared to the Zn (002) crystal facet, the Zn (101) facet shows a larger electron accumulation and a more delocalized charge density difference distribution at the interface, suggesting that additive molecules are more strongly adsorbed over Zn (101) facet driven by increased Zn–H interactions (Fig. S17). These results clearly highlight the strong influence of the chemical moieties surrounding the electron-donating center on the binding affinities to the zinc surface. Notably, HMPA and TMPA exhibit stronger binding to the high-energy, unstable (101) facet compared to PA, which can be attributed to the presence of methyl groups enhancing surface interactions. This trend is in-line with the higher (002)/(101) intensity ratios observed in XRD (Fig. 5B), following the order: HMPA > TMPA > PA > bare electrolyte. Preferential binding to the (101) facet facilitates its transformation into the thermodynamically stable (002) facet, resulting in more uniform, epitaxial zinc deposition. This correlates well with the improved deposition morphology and stability observed earlier, particularly in the case of HMPA. Surface reconstruction in metal anodes is often inferred primarily from computational modeling. This underscores atomic-scale insight into interfacial rearrangements and facet-dependent stability. However, only a limited number of experimental studies directly discuss such reconstruction. Cui *et al.* deduced their electrolyte reconstructs the surface of the Zn anode to generate a robust solid electrolyte interface (SEI), which promotes uniform zinc deposition.^63^ Additionally, we experimentally investigated the surface reconstruction phenomenon by depositing at different capacities and measuring the Zn (002) facet intensity, as shown in Fig. S18. With increasing capacity, the Zn (002) facet intensity increases, underscoring that prolonged electrodeposition time makes the surface reconstruction more apparent.

## Conclusion

This study reveals the essential role of interfacial interactions in controlling the performance and stability of zinc metal anodes in aqueous zinc metal batteries. By examining amide-based additives with subtle molecular differences, we show how these molecules influence solvation and surface adsorption to modify charge transfer kinetics and zinc electrodeposition behavior. For the first time, we establish kinetic reversibility, defined by the width of the steady state kinetic regime, as a valuable metric alongside exchange current density to comprehensively evaluate anode stability. Our findings are supported by fast scan voltammetry on ultramicroelectrodes, density functional theory calculations, and thorough electrochemical and structural analyses. The exceptional improvements observed with trace additive concentrations originate from enhanced interfacial stabilization that drives kinetic irreversibility. This kinetic effect governs electrode performance regardless of the relative contributions of solvation or adsorption. This work advances the fundamental understanding of additive–electrode interactions and provides a robust framework for designing electrolyte additives and formulating novel electrolytes to enhance anode stability. Although demonstrated on zinc anodes, the findings are rooted in fundamental electrochemical principles and are expected to be broadly applicable to other metal battery chemistries, including lithium and sodium.

## Author contributions

The concept of the paper was conceived by B. D., A. B., and J. E. D. All electrochemical experiments were done by J. H. N. and A. R. DFT were done and analyzed by B. R. B and S. C. S. P. did the electronic microscopic characterization. N. R. and I. D. synthesized and analyzed the molecules for this study. J. H. N., A. R., K. S., B. R. B, B. D., A. B., and J. E. D. helped in the interpretation of the data. All authors have agreed to the final version of the manuscript.

## Conflicts of interest

There are no conflicts to declare.

## Supplementary Material

SC-OLF-D5SC06311F-s001

## Data Availability

Raw data that support the findings of this study are available from the corresponding author, upon reasonable request. The authors confirm that the data supporting the findings of this study are available within the article and its supplementary information (SI). Supplementary information: synthesis procedures, optimized structures, binding energies, cyclic voltammetries, X-RayCT, and ECMS data. See DOI: https://doi.org/10.1039/d5sc06311f.
