# Supplementary material for: Amide additives enhance the understanding of kinetic reversibility in zinc anode stability using ultramicroelectrodes
Source: Chem Sci. 2025 Oct 31;17(4):2302–16. doi: 10.1039/d5sc06311f (PMC12679528; doi:10.1039/d5sc06311f)
Supplement: SC-017-D5SC06311F-s001 [file SC-017-D5SC06311F-s001.pdf]

Supplementary Information (SI) for Chemical Science.
This journal is © The Royal Society of Chemistry 2025

Supporting Information

Amide Additives Enhance the Understanding of Kinetic Reversibility in Zinc Anode Stability Using Ultramicroelectrodes

James H. Nguyen[a,£], Ashutosh Rana[a,£], Kudekallu Shiprath[b, £], Brajesh R. Bhagat[b, £], Saptarshi Paul[a], Shaonsikta Chatterjee[b], Newton Roy[b], Ishita Das[b], Bidisa Das[b,c], Abhik Banerjee[b,c], Jeffrey E. Dick[a,d]

[a] Department of Chemistry, Purdue University, West Lafayette, IN, 47907, USA
[b] Research Institute for Sustainable Energy, Center for Research and Education in Science and Technology (TCG-CREST), Salt Lake, Kolkata 700091, India
[c] Academy of Scientific and Innovative Research (AcSIR), Ghaziabad 201002, India
[d] Elmore Family School of Electrical and Computer Engineering, Purdue University, West Lafayette, IN, 47907, USA

*Corresponding Authors
Jeffrey E. Dick (jdick@purdue.edu)
Abhik Banerjee (abhik.banerjee@tcgcrest.org)
Bidisa Das (bidisa.das@tcgcrest.org)
£: Equal Contribution

Experimental Section:

1. Materials

Zinc Triflate ($Zn(OTf)_2$, of reagent grade) was acquired from Sigma Aldrich. Zinc foil (99.99% purity) was obtained from MTI Corporation. These chemicals were employed as received without further purification. Whatman glass microfiber separators were purchased from Sigma Aldrich. Deionized (DI) water was acquired from a Milli-Q ultrapure water production system and employed in the formulation of all aqueous electrolytes. The Ag/AgCl electrode in saturated KCl and the Pt counter electrode were procured from CH Instruments.

2. Preparation of the Electrolyte and Electrode

Electrolytes were formulated by dissolving 1 M $Zn(OTf)_2$ in deionized (DI) water at room temperature (25°C), supplemented with 0.3 M HMPA, 0.3 M TMPA, and 0.015M PA additive. Zinc foils were converted into discs and utilized as Zn electrodes. Zinc foils were used as the electrodes for symmetric cell and asymmetric cell measurements.

3. Material Characterization

Electrochemical measurements were conducted in conjunction with in situ optical microscopy using an Olympus optical microscope connected to a CHI potentiostat in a 3-electrode setup. A customized optical cell, featuring a specially designed 1mm Cu macroelectrode, was utilized for the electrochemical experiments. Zinc and Ag/AgCl were employed as the counter and reference electrodes, respectively. Additionally, electrochemical mass spectrometry was carried out using a system provided by Spectro Inlets ApS, which interfaces an electrochemical cell with a mass spectrometer through a semipermeable hydrophobic microchip.

4. Electrochemical Measurements

All electrochemical experiments were conducted utilizing a CHI6284 electrochemical workstation. CV and LSV measurements were carried out in a three-electrode configuration as detailed in the main manuscript. CR2032 coin-type cells were assembled under ambient air conditions and evaluated using a Neware battery test system. Symmetric cells were fabricated employing two Zn foils (with a diameter of 15 mm and thickness of 25 μm), a glass microfiber separator (with a diameter of 19 mm), and 50 μL of electrolyte. Galvanostatic charge-discharge cycles were performed for the symmetric cell test at a current density of 1 mAg-1 , with a capacity of 0.5 mAhg-1 being deposited. All electrochemical tests were conducted at 25°C under standard conditions

5. Computational Analysis

The studies for small molecules and clusters were performed using density functional theory (DFT) as implemented in the GAUSSIAN 16, suite of *ab initio* quantum chemistry program.[1] Geometry optimizations and vibrational frequency calculations were done using both hybrid

B3LYP[1–4] and M05-2X[5] functionals with the double-zeta quality 6-31+G** basis set for all atoms. The default SCF and geometry convergence criteria were used, and no symmetry constraints were imposed for the structural optimizations. Harmonic frequency analysis was employed to characterize the stationary points as stable structures. We found very similar structural parameters using both methods and the trends in calculated free energies were identical, however, we have reported the Gibb's free energies calculated using M05-2X in the text. The calculated dipole moments, electronegativities are presented in the Supporting Information were calculated using B3LYP functional. The electronegativity has been calculated from the energies of highest occupied molecular orbital (HOMO) and lowest unoccupied molecular orbital (LUMO) using the expression[6]: Electronegativity (χ) = (IP+EA)/2, where IP and EA are the ionization potential and electron affinity of any chemical system. IP and EA can be approximately obtained from calculated HOMO and LUMO energies. We have used Grimme's D3 dispersion correction for all our solution state studies.[7]

For any chemical reaction, Reactants → Products, the Gibb's free energy of reaction is calculated as $\Delta G_{reaction} = \Sigma_{products}G - \Sigma_{reactants}G$, where G represents the sum of electronic and thermal free energies of the corresponding species. To model the aqueous phase, we have conducted single point solvent phase studies on the optimized structures using density-based model (SMD)[8,9] using the self-consistent reaction field (SCRF) method employing implicit solvation with water as a solvent (dielectric constant, ε = 78.39). The free energies (G_w) in aqueous phase are then obtained directly from the optimization of the molecules in water medium. In case of Zn_4 cluster the binding energy is calculated using the following equation: $E_{bind} = E_{Zn_4\text{-adsorbate}} - (E_{Zn_4} + E_{adsorbate})$ where $E_{Zn_4\text{-adsorbate}}$ represents the energy of the Zn cluster with additive, triflate ion, water or OH^- ion bound to it, the energy of the Zn_4 cluster is E_{Zn_4} and $E_{adsorbate}$ gives the energy of the free neutral or negative ion, respectively.

The binding energy calculations on the zinc surface were all performed using DFT as implemented in the QuantumATK 2018.06 software.[10] In the present study, the exchange correlation functional is approximated by the generalized gradient approximation (GGA) and the SG15 optimized norm-conserving pseudopotentials were used for the atoms with numerical atomic orbital basis sets of high accuracy. The density mesh cut-off for plane-wave expansion is set to be 160 Ry for the crystalline surfaces. For the calculation of binding energies of various neutral molecules on Zn surface, a supercell of 4 × 4 × 1 for Zn(002), and 4 × 2 × 1 for Zn(101) atoms with a (002/101) upper surface was made with a vacuum gap of 20 Å on top. A Monkhorst Pack grid of 4 × 4 × 1, with force convergence criterion of 0.02 eV/Å has been used for the self-consistent field cycle calculations. After binding, the surface of the supercell is optimized (top two layers) keeping a few lower layers fixed. The optimized surface shows the correct conformation of the molecules/ions adsorbed along with the adsorption sites.

The surface energy (E_{surf}) of the Zn(002), and Zn(101) surface is calculated using, $E_{surf} = (E_{slab} - n \times E_{bulk})/A$, where E_{slab} is the energy of the slab, n is the number of atoms in the slab, E_{bulk} is the energy per atom of bulk structure, and A is surface area of the slab. The binding energy (E_{bind}) of the molecules to the Zn(002/101) surface is calculated using $E_{bind} = E_{Zn\text{-adsorbate}} - (E_{Zn(002/101)} + E_{adsorbate})$ where $E_{Zn\text{-adsorbate}}$ represents the energy of the Zn binding surface with (002/101) face on the top and the respective neutral additive molecule bound to it, the energy of the pristine Zn surface is $E_{Zn(002/101)}$ and $E_{adsorbate}$ gives the energy of the free neutral molecule, respectively.

6. Synthesis of Phosphoramide and Trimethylphosphoramide

Synthesis Procedure of PA:

To a stirred solution of liquid ammonia (NH_3) at -78°C, was added $POCl_3$ (3 g, 1 eq) dropwise dissolved in dry THF (30 ml). The reaction mixture was stirred for 45 minutes at -78°C. Then the reaction mixture again stirred for 4h at ambient temperature, white suspension was obtained. the reaction mixture transferred into a single neck RB, concentrated the reaction mixture under argon atmosphere to get the crude (5.2 g) material (white powder). Added diethyl amine to the crude material and the resulting mixture was refluxed for 3h. The reaction mixture was cooled to ambient temperature, decan excess amount of diethyl amine, the white solid material was washed with chloroform (3 times), concentrated the material to get the desired product (phosphoramide) as white solid. Yield: 1.52 g (82%).

Synthesis Procedure of TMPA:

Phosphoryl chloride (5.11 g, 33.3 mmol) was added dropwise to an excess of methylamine (2M in THF) (101ml, 202.61mmol) in hexane (250 mL) at 0°C, a white precipitate formed. Warmed the solution slowly to 23°C and stirred the solution for 2 hours. Removed the hexane in vacuum and added chloroform (250 ml) to the sticky white solid. Filtered the methylammonium chloride on a Buchner funnel and was added a potassium hydroxide/methanol solution dropwise until pH > 7. Removed the potassium chloride by filtration and reduced the volume of the solution to 50 ml and was added diluted hydrochloric acid (2 M) to the solution until pH = 5-6. To this solution DCM (150 ml) was added then filtered the solution and reduced the volume of the filtrate to 50 ml. Removed the DCM in vacuum at 40°C and finally washed with hexane (10 ml) to the concentrated solution and get off white solid powder like product. Yield: 3.03 g (66%).

a

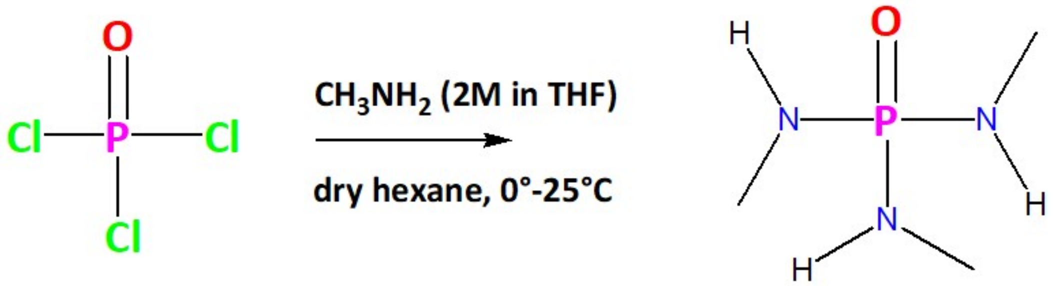

O
Cl
P
Cl
Cl
CH3NH2 (2M in THF)
dry hexane, 0°-25°C
H
O
N
P
N
H
N
H

b

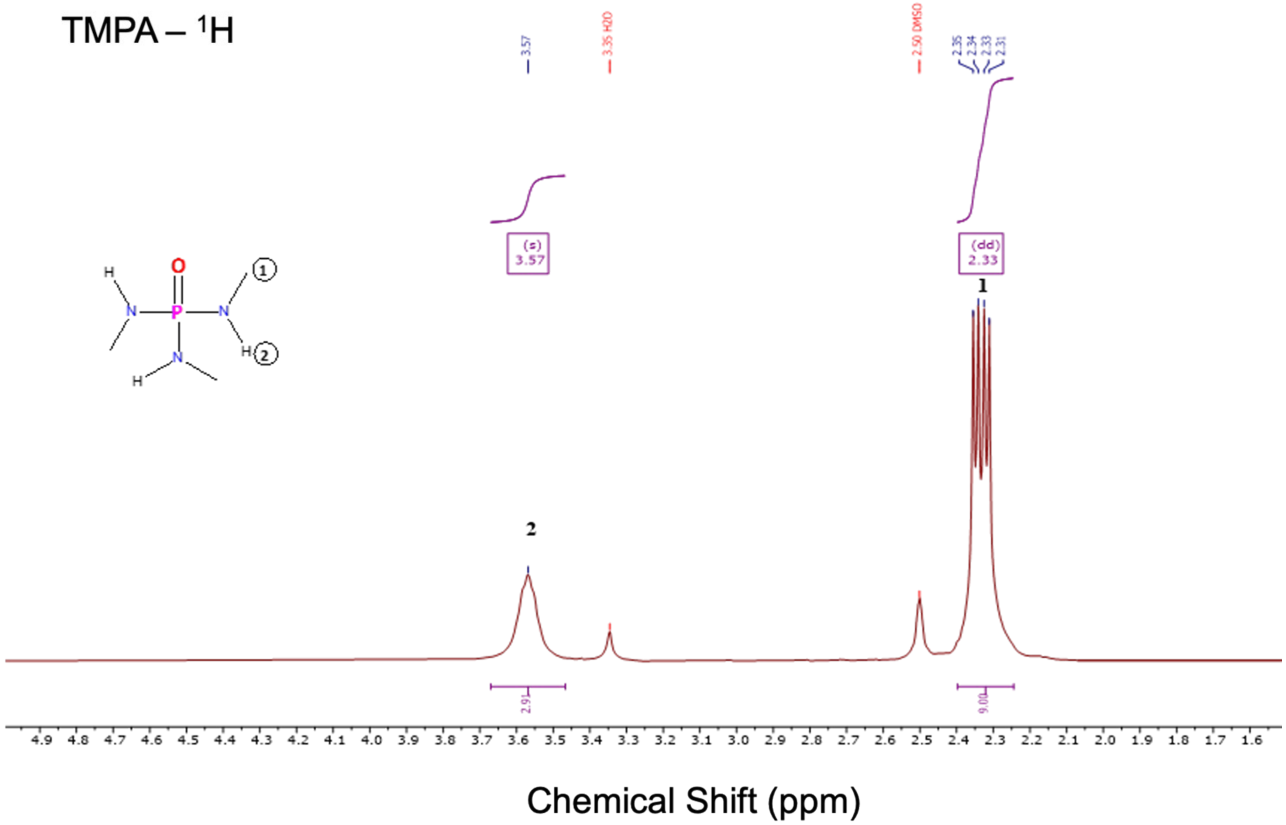

TMPA – 1H
3.57
3.35 H2O
2.50 DMSO
2.35
2.34
2.33
2.31
(s)
3.57
(dd)
2.33
1
2
2.91
9.00
4.9 4.8 4.7 4.6 4.5 4.4 4.3 4.2 4.1 4.0 3.9 3.8 3.7 3.6 3.5 3.4 3.3 3.2 3.1 3.0 2.9 2.8 2.7 2.6 2.5 2.4 2.3 2.2 2.1 2.0 1.9 1.8 1.7 1.6
Chemical Shift (ppm)

c

TMPA – ^{1}H

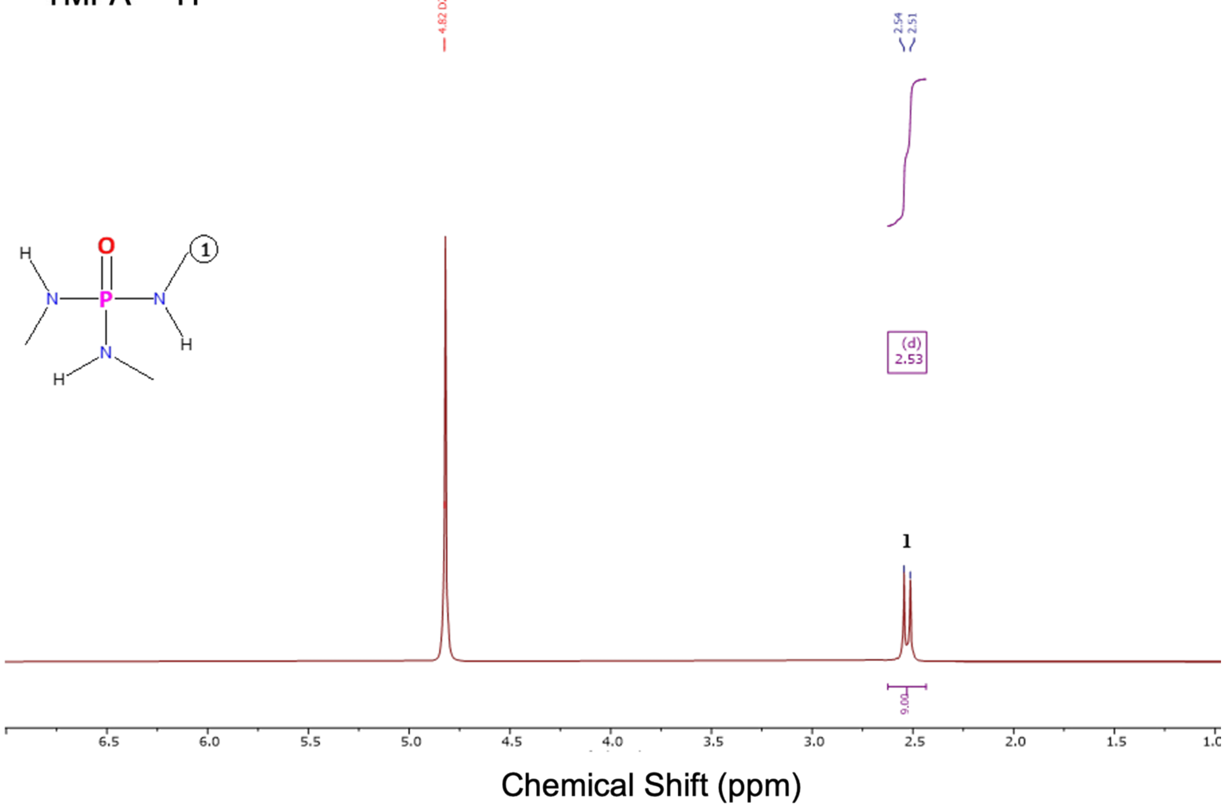

4.82 D2O
2.54
2.51
(d)
2.53
1
9.00
6.5
6.0
5.5
5.0
4.5
4.0
3.5
3.0
2.5
2.0
1.5
Chemical Shift (ppm)

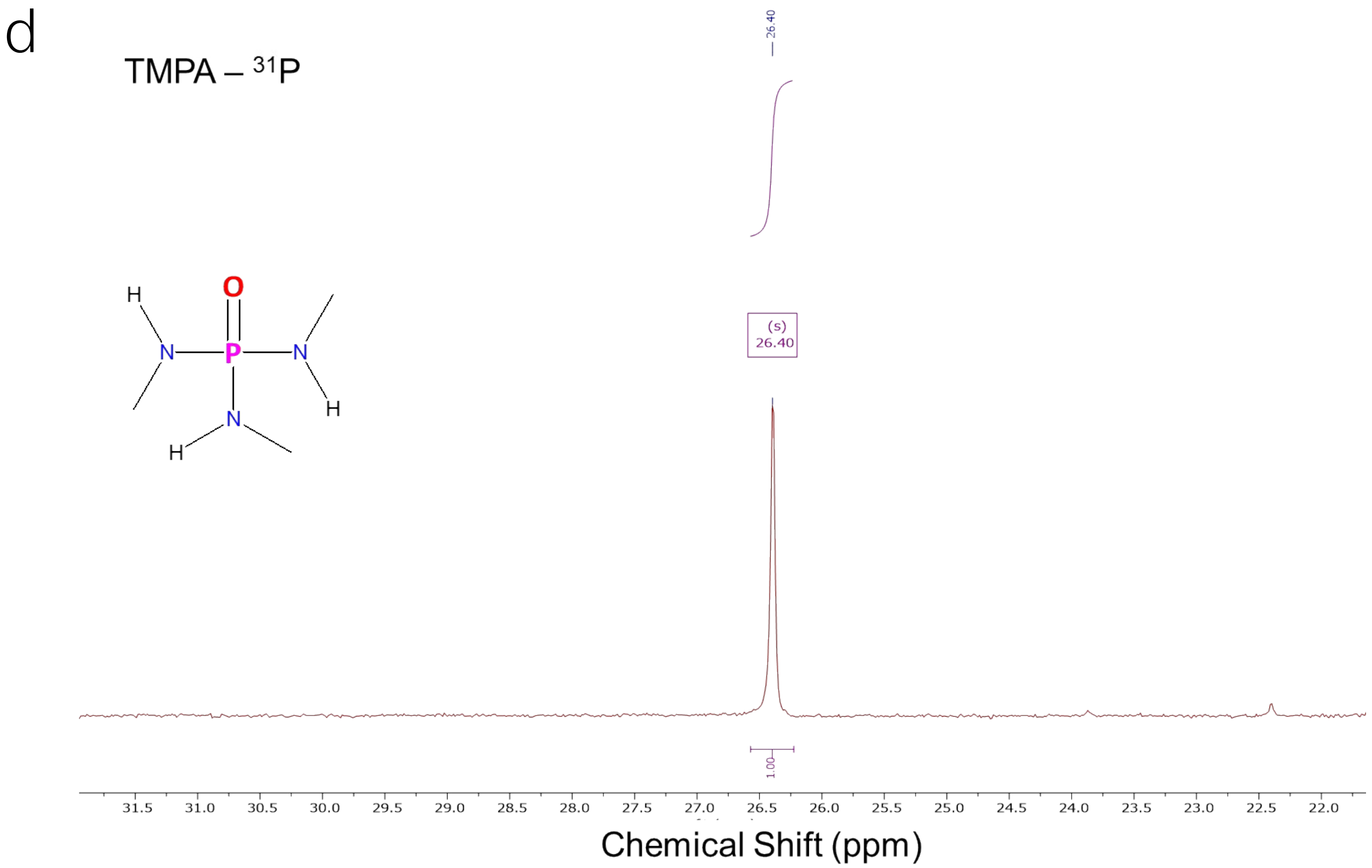

Figure S1. (a) Synthesis scheme of TMPA. (b) ^{1}H NMR of TMPA (400 MHz, d^6-DMSO, δ/ppm): 3.57 (s, 3H), 2.35-2.31 (dd, 9H). (c) ^{1}H NMR of TMPA (400 MHz, D_2O, δ/ppm): 2.54-2.51 (d, 9H) (d) ^{31}P NMR TMPA (162 MHz, D_2O, δ/ppm): 26.40 (s).

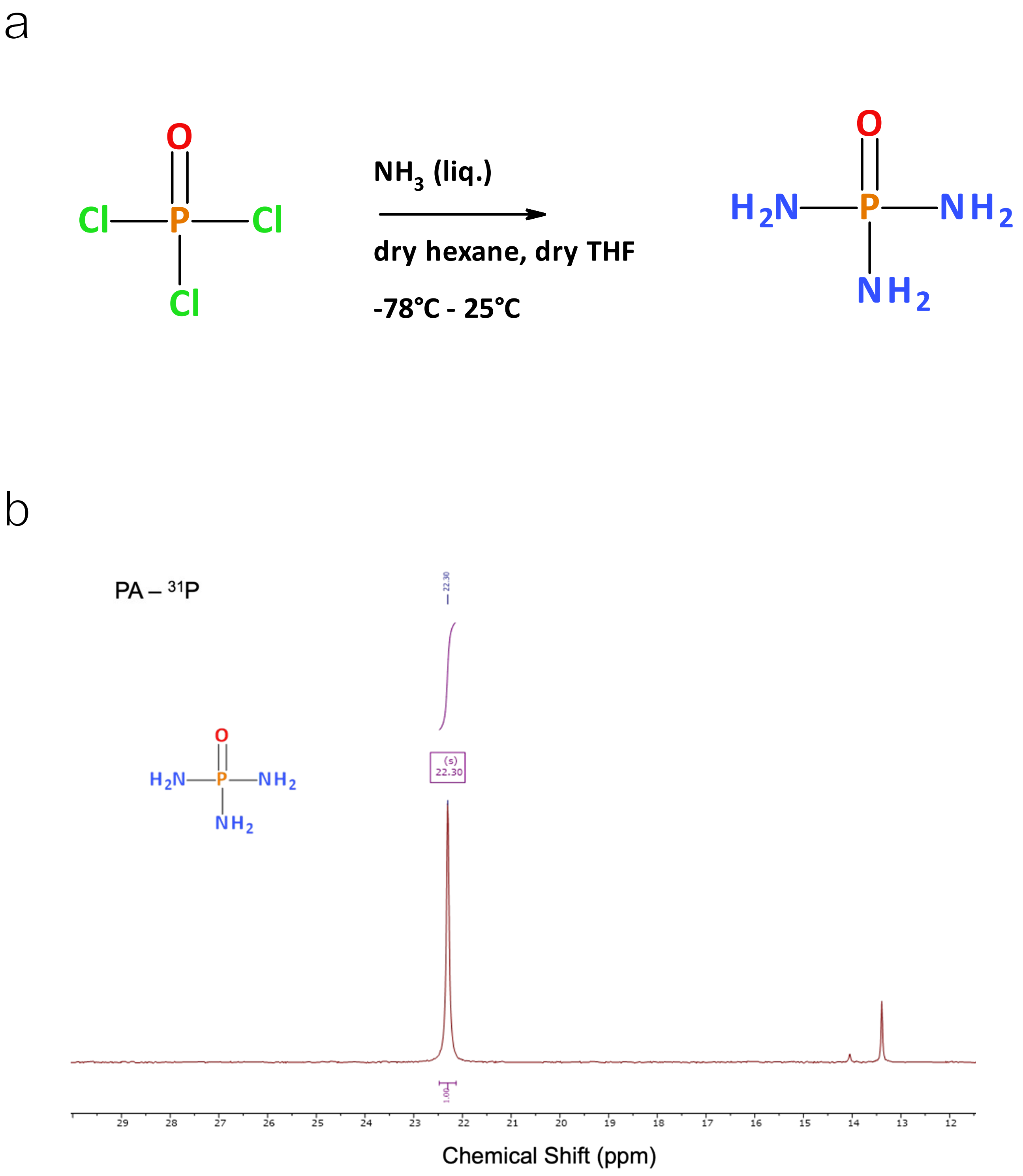

Figure S2. (a) Synthesis scheme of PA. (b) ^{31}P NMR of PA (162 MHz, D_2O, δ/ppm): 22.30 (s).

Table S1. Dipole moments, frontier molecular orbitals and electronegativities of the additives.

	**Dipole moment (Debye)**	**HOMO (eV)**	**LUMO (eV)**	**Electronegativity**
HMPA	3.95	-6.00	-0.18	3.09
TMPA	4.10	-6.50	-0.39	3.44
PA	4.32	-7.34	-0.62	3.98

Table S2. Comparison of the OTf^- binding to hydrated Zn ion, $[Zn(H_2O)_6]^{2+}$ in monodentate and bidentate form, structures and Gibb's free energies in solution. In monodentate binding the ligand is anchored by only one bond, while in bidentate binding the anchorage is via two bonds.

**Reactions**	**ΔG_w (kcal/mol)**	**Optimized structures of Zn complexes**
Monodentate binding<br><br>$[Zn(H_2O)_6]^{2+} + OTf^-$<br>$\rightarrow [Zn(H_2O)_3(OTf)]^+ + 3H_2O$	-6.65	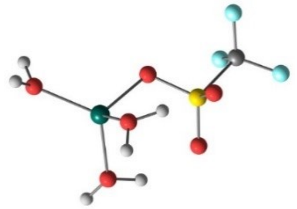
Bidentate binding<br><br>$[Zn(H_2O)_6]^{2+} + OTf^- \rightarrow$<br>$[Zn(H_2O)_2(OTf)]^+ + 4H_2O$	-3.21	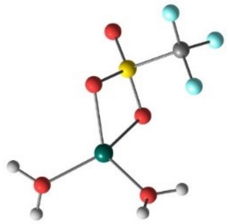

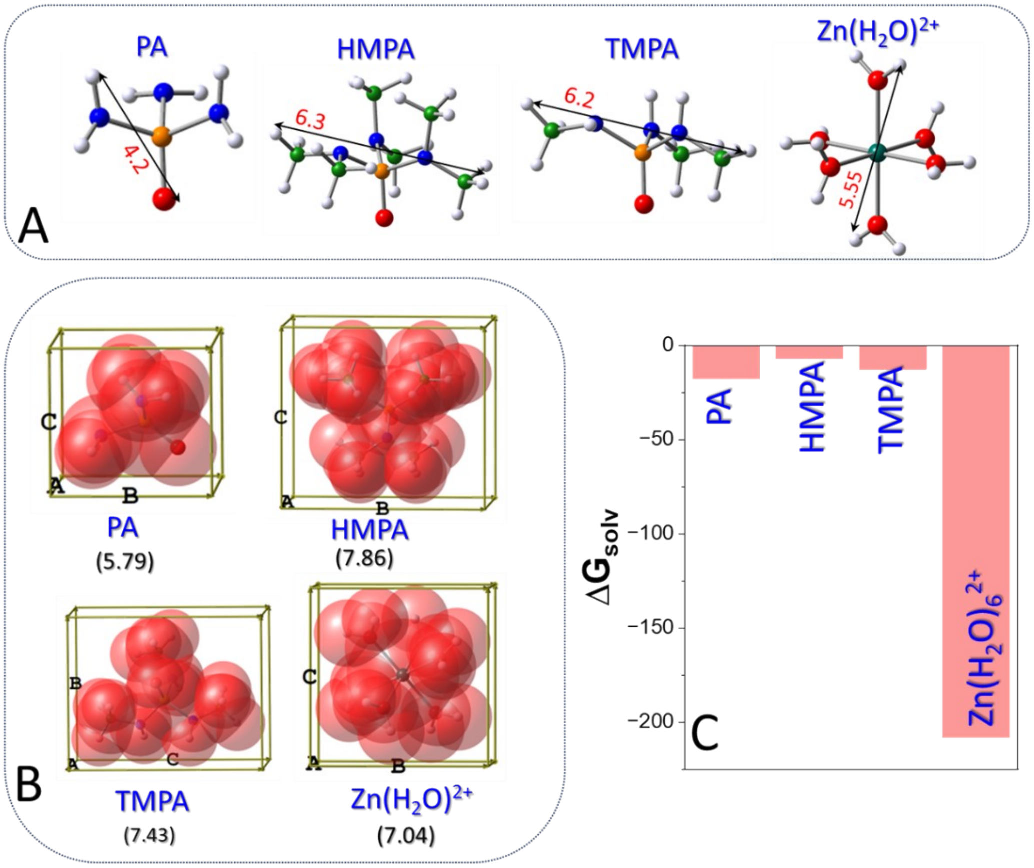

Figure S3. **A.** Optimized structures along with longest end-to-end distances (Å) of additives and $Zn(H_2O)_6^{2+}$ ion. **B.** the calculated collision diameter (Å) of the corresponding species written within the parentheses **C.** the Gibb's free energy of solvation for PA, HMPA, TMPA, and $Zn(H_2O)_6^{2+}$ ion. Color code: Grey balls: C, white small balls: H, Red balls: O, Dark Blue balls: N, Orange balls: P, Yellow balls: S, Cyan Balls: F, Greyish green balls: Zn.

Table S3. Calculated reaction free energies of ligand transfer reactions starting from $[Zn(H_2O)_6]^{2+}$ and $[Zn(H_2O)_2(OTf)]^{+}$ in aqueous medium. 'L' stands for ligand, the additive molecules, PA, TMPA and HMPA. In monodentate binding the ligand is anchored by only one bond, while in bidentate binding the anchorage is via two bonds.

**Reactions (aqueous)**	**ΔG_w(kcal/mol)**		
	**L= HMPA**	**L= TMPA**	**L=PA**
$[Zn(H_2O)_6]^{2+} + L = [Zn(H_2O)_3L]^{2+} + 3H_2O$	-16.36	-14.14	-14.86
$[Zn(H_2O)_3(OTf)]^{+} + L = [Zn(H_2O)_2(OTf)L]^{+} + H_2O$ **(monodentate)**	-7.93	-7.74	-5.58
$[Zn(H_2O)_2(OTf)]^{+} + L = [Zn(H_2O)(OTf)L]^{+} + H_2O$ **(bidentate)**	-14.17	-11.28	-11.41

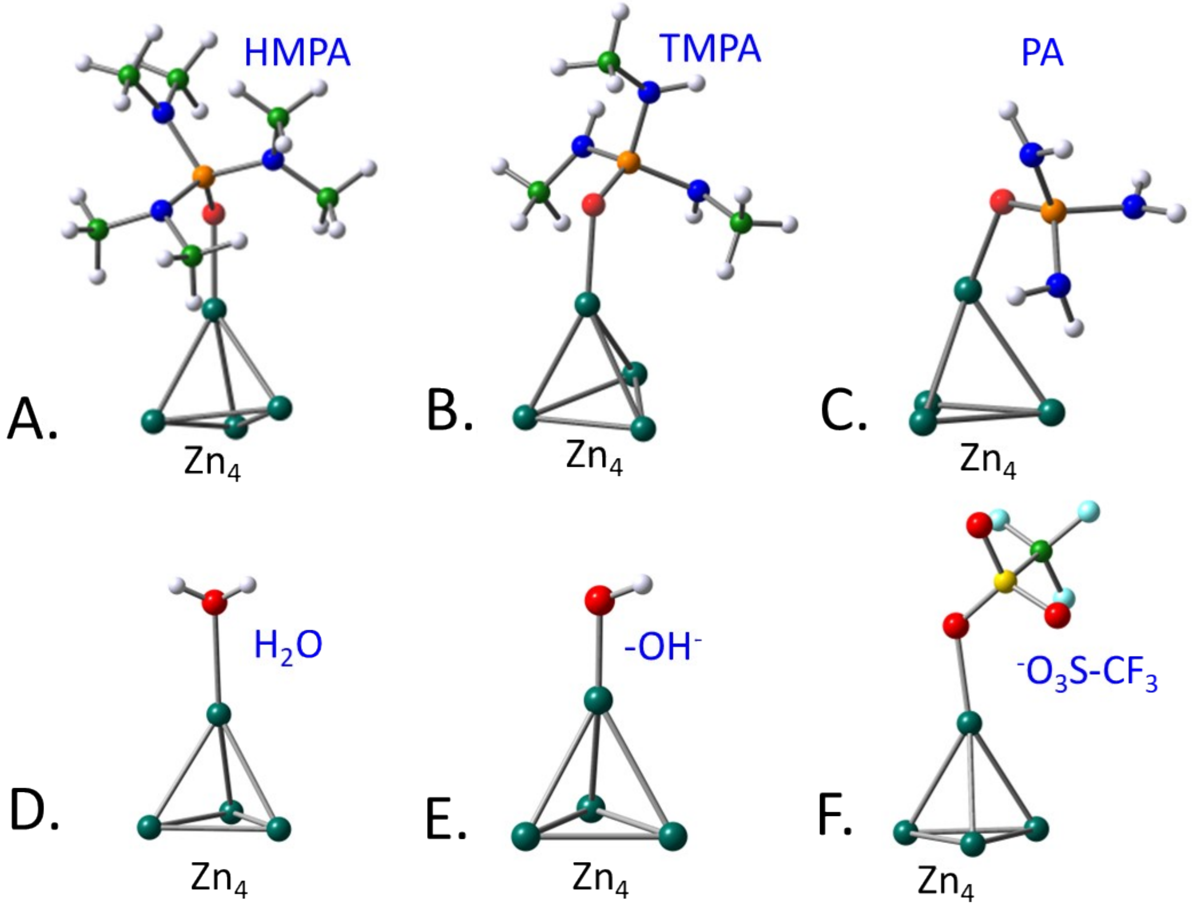

Figure S4. Optimized structures of additive and ion binding on neutral Zn_4 cluster.

Table S4. Calculated binding energies of the additive molecules and other ligands on Zn_4 cluster in gas phase.

**Species binding to Zn_4 Cluster**	**ΔE(kcal/mol)**
HMPA	-14.46
TMPA	-13.79
PA	-12.17
water	8.47
OH^-	-71.28
OTf^-	-21.12

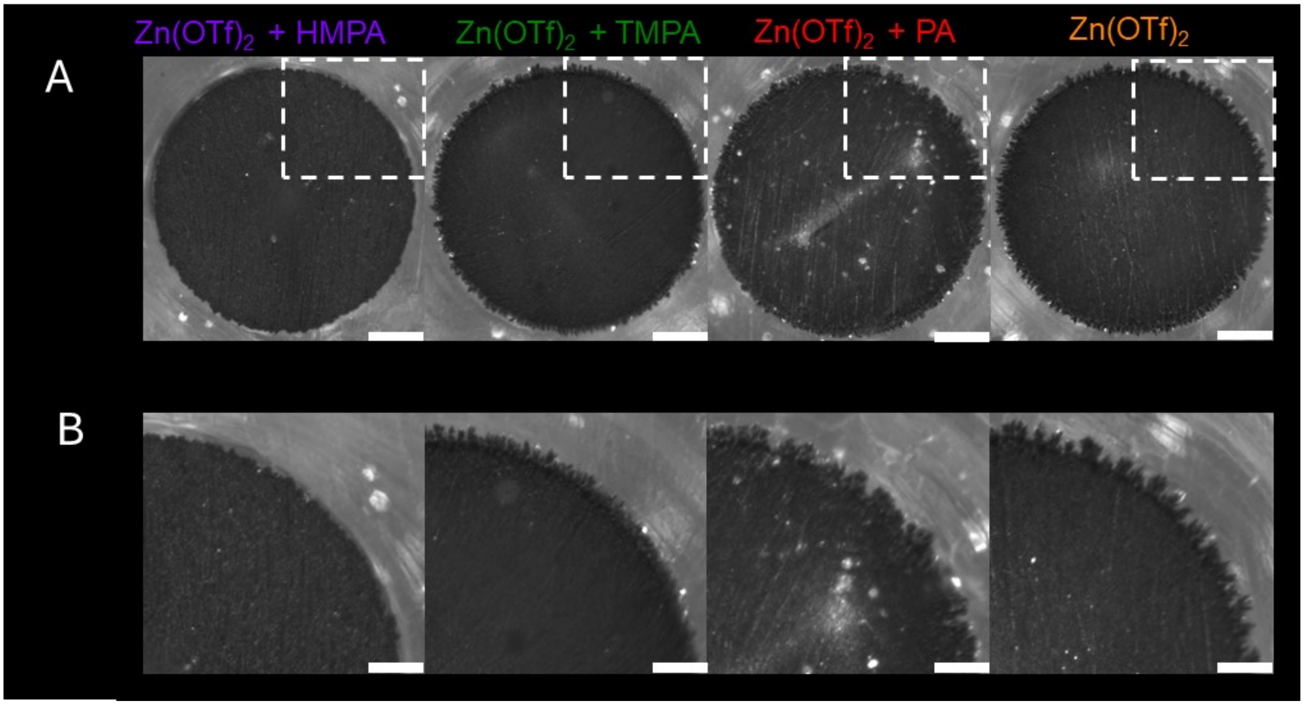

Figure S5. (A) In situ optical microscopy snapshots at -1.6 V during the voltammetric sweep for different electrolytes. Scale bar is 250 μm. (B) In situ optical microscopy snapshots of peripheral of Cu disk showing dendritic growth morphology. Scale bar is 125 μm.

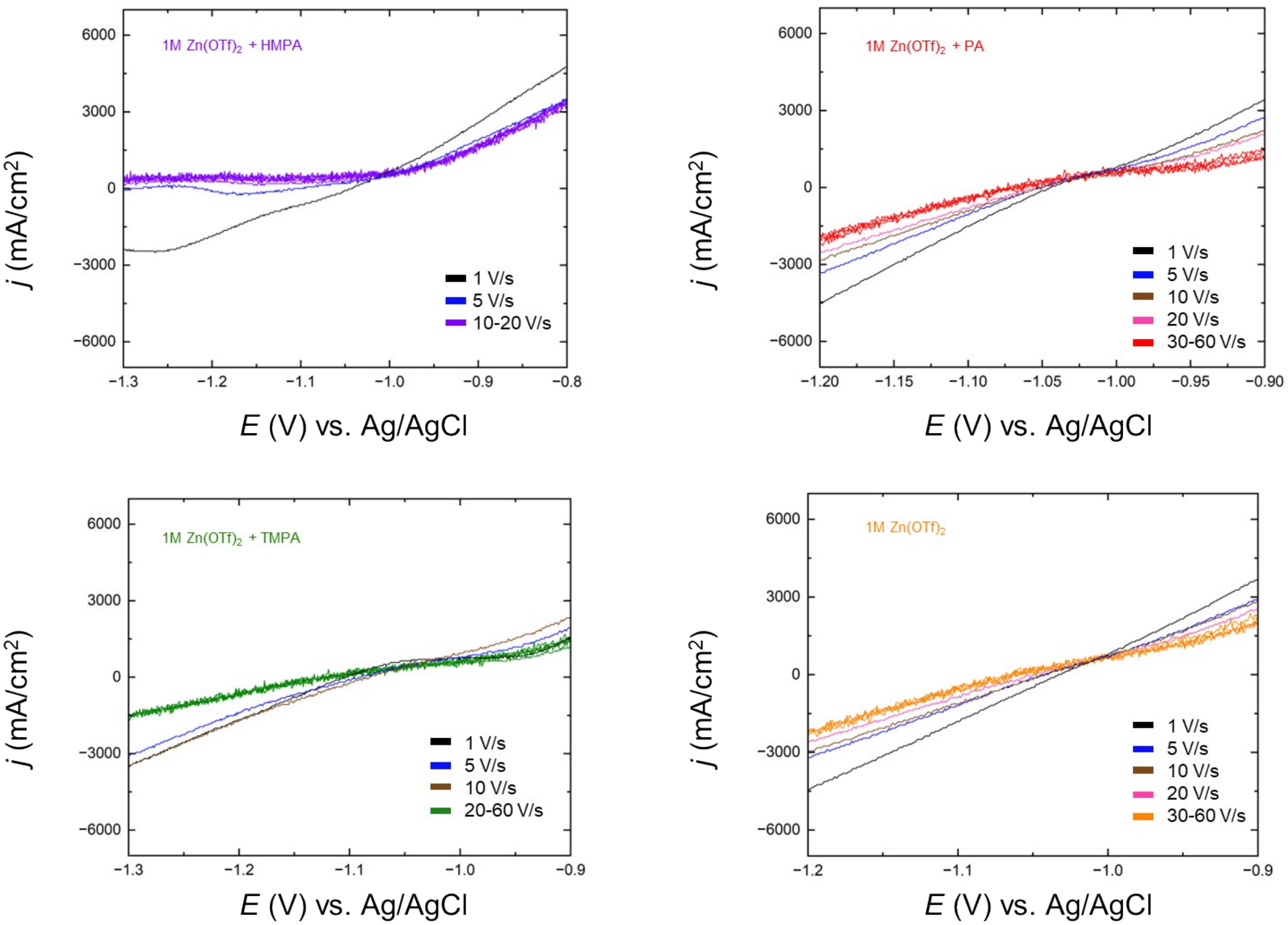

Figure S6. FSCV showing kinetic regime convoluted by mass transfer at slower scan rates for all the electrolytes.

HMPA slow scans are at 1 and 5 V/s. TMPA slow scans are at 1, 5, and 10 V/s. PA and bare electrolyte slow scans are at 1, 5, 10, 20 V/s. The slow scans can be shown to deviate from the kinetic regime.

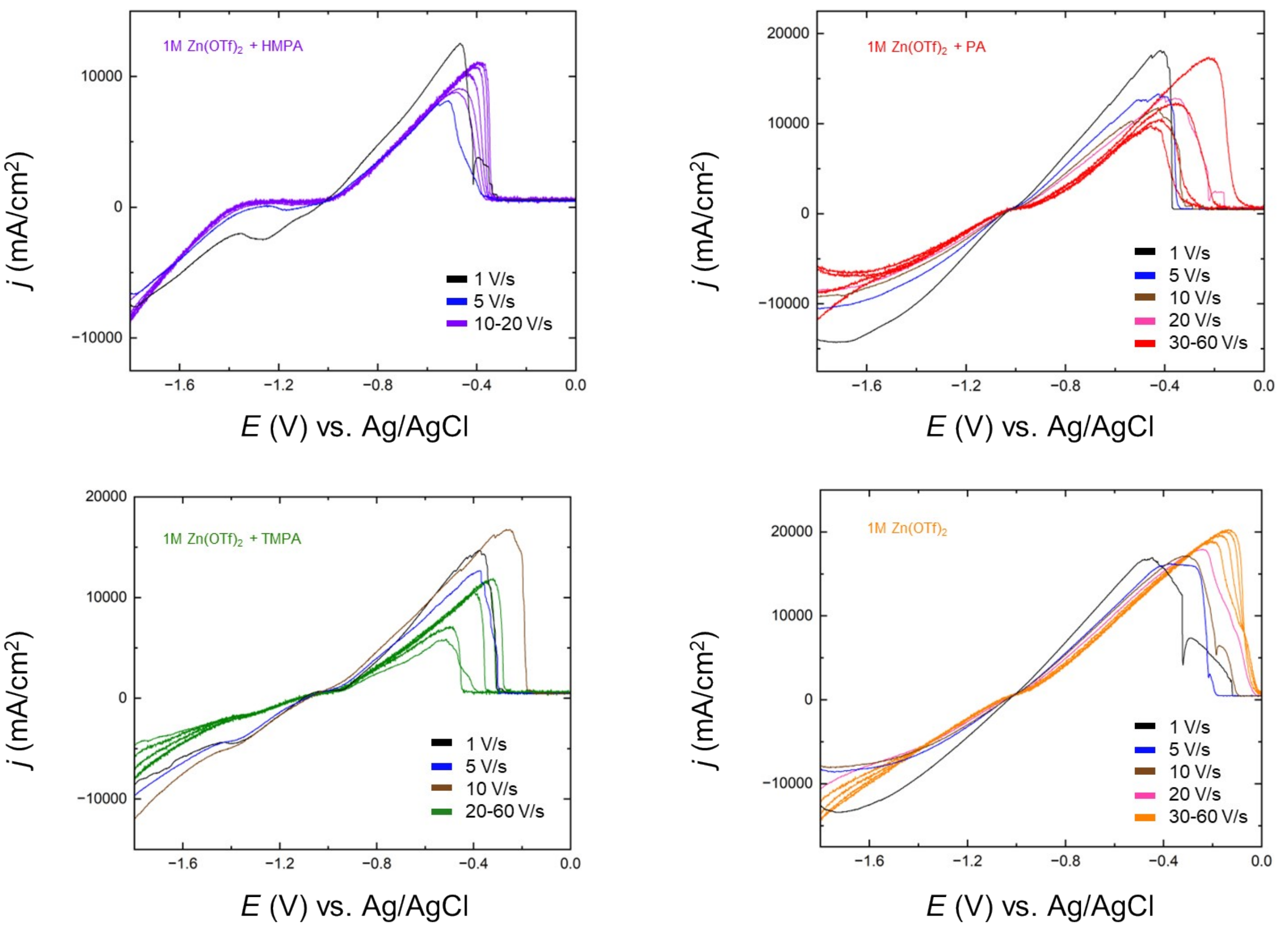

Figure S7. FSCV showing the “cut-off” scan rate for all the electrolytes.

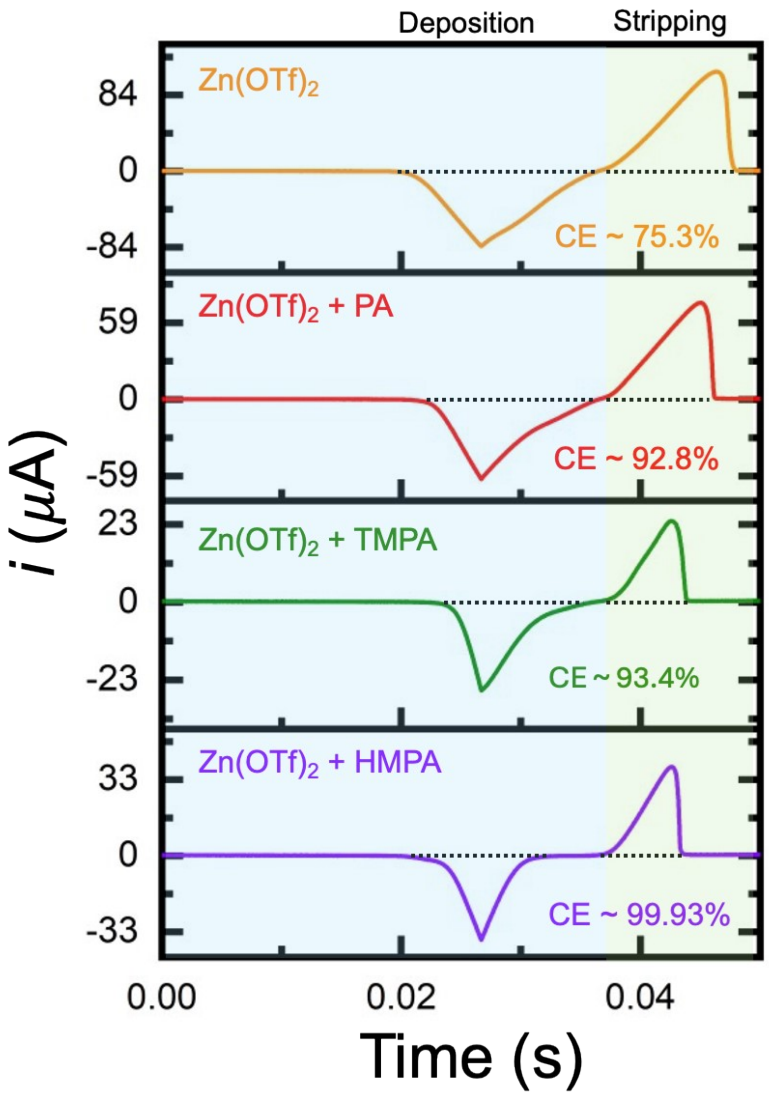

Figure S8. Coulombic efficiency for all the electrolytes at 60 V/s.

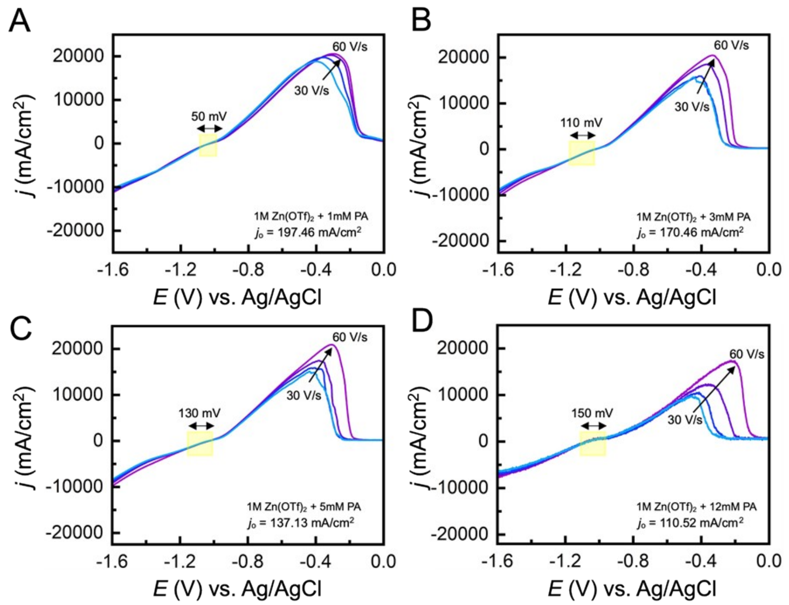

Figure S9. (A) Backward sweep of the fast scan cyclic voltammograms with kinetically controlled low-overpotential regimes as a function of scan rates for 1 mM PA in 1M $Zn(OTf)_2$, showing the width of kinetic regime as well as the exchange current density for the voltammogram at 60 v/s (B) Same as panel A but for 3 mM PA in 1M $Zn(OTf)_2$ (C) Same as panel A but for 5 mM PA in 1M $Zn(OTf)_2$ (D) Same as panel A but for 12 mM PA in 1M $Zn(OTf)_2$

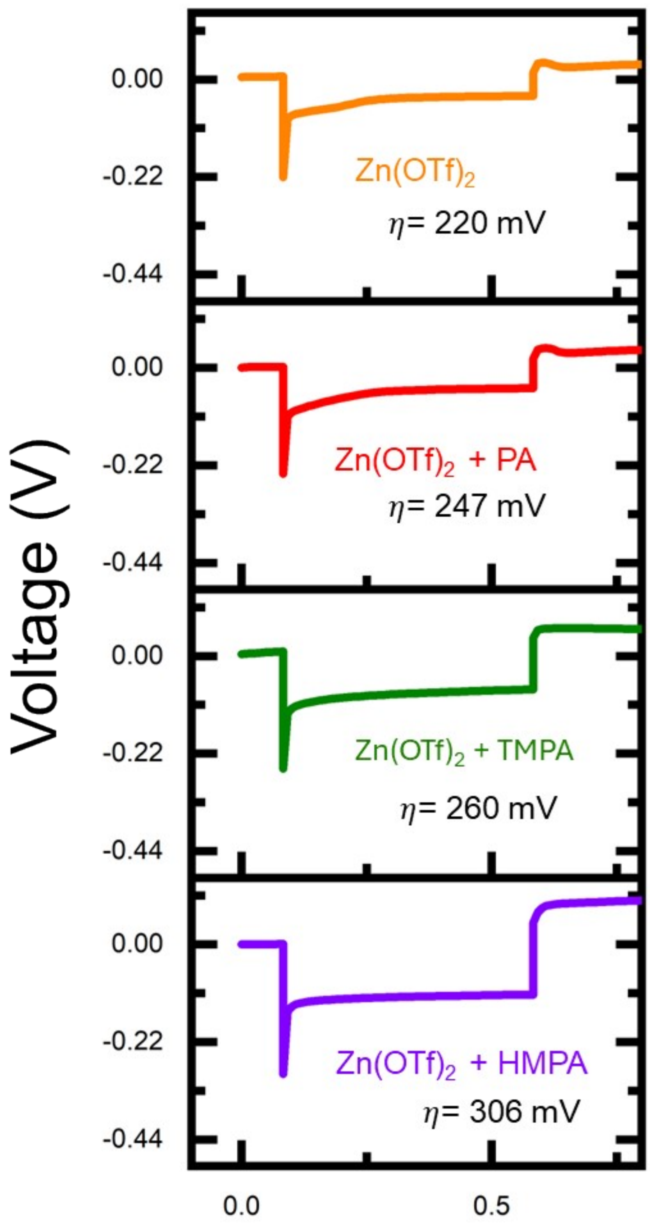

Figure S10. Measured nucleation overpotential from Zn|Zn symmetric cell for all the electrolytes.

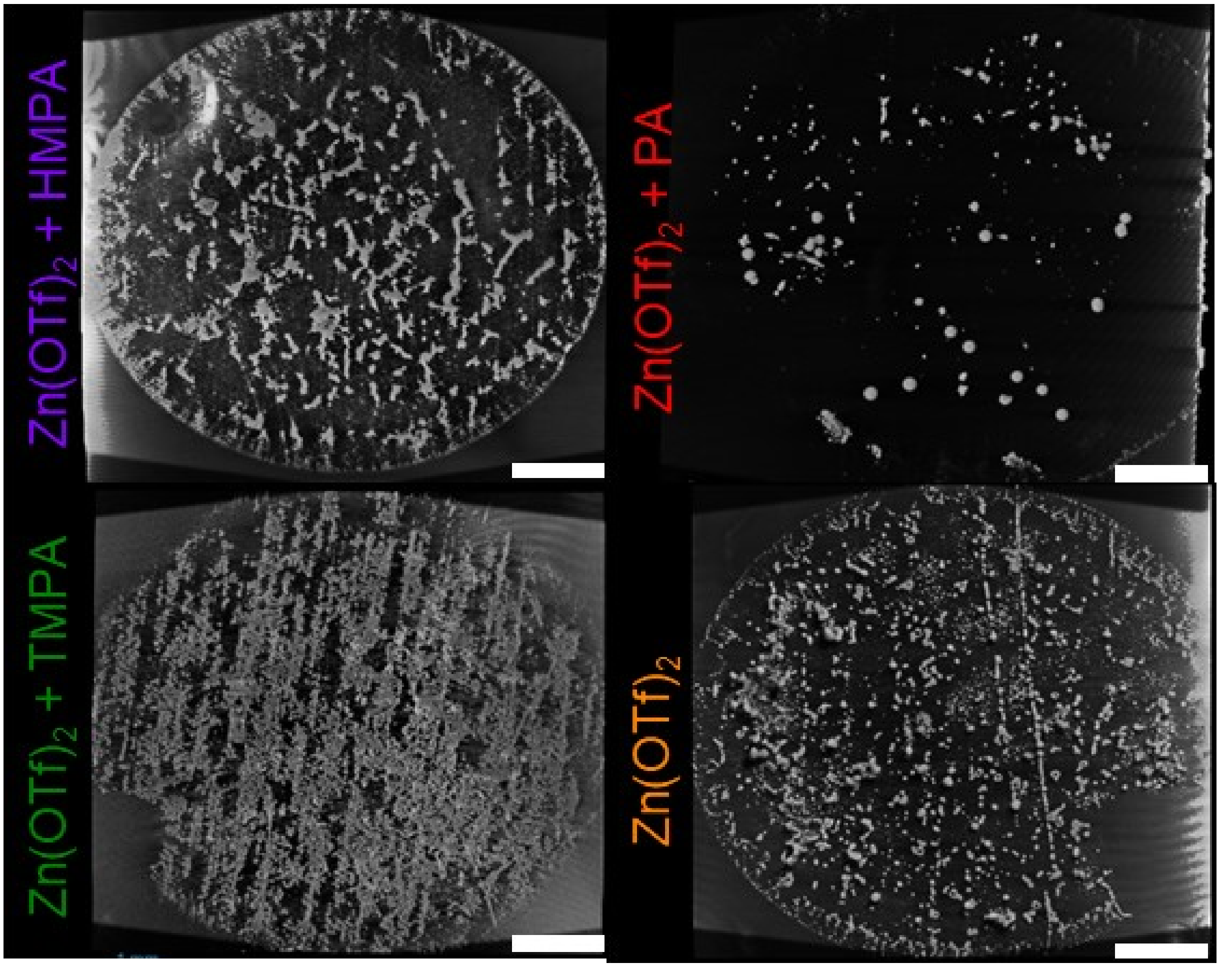

Figure S11. Xray-CT for all the electrolytes showing deposition at 2.5 mA/cm^2 (5 mAh/cm^2). The scale bar is 1 mm.

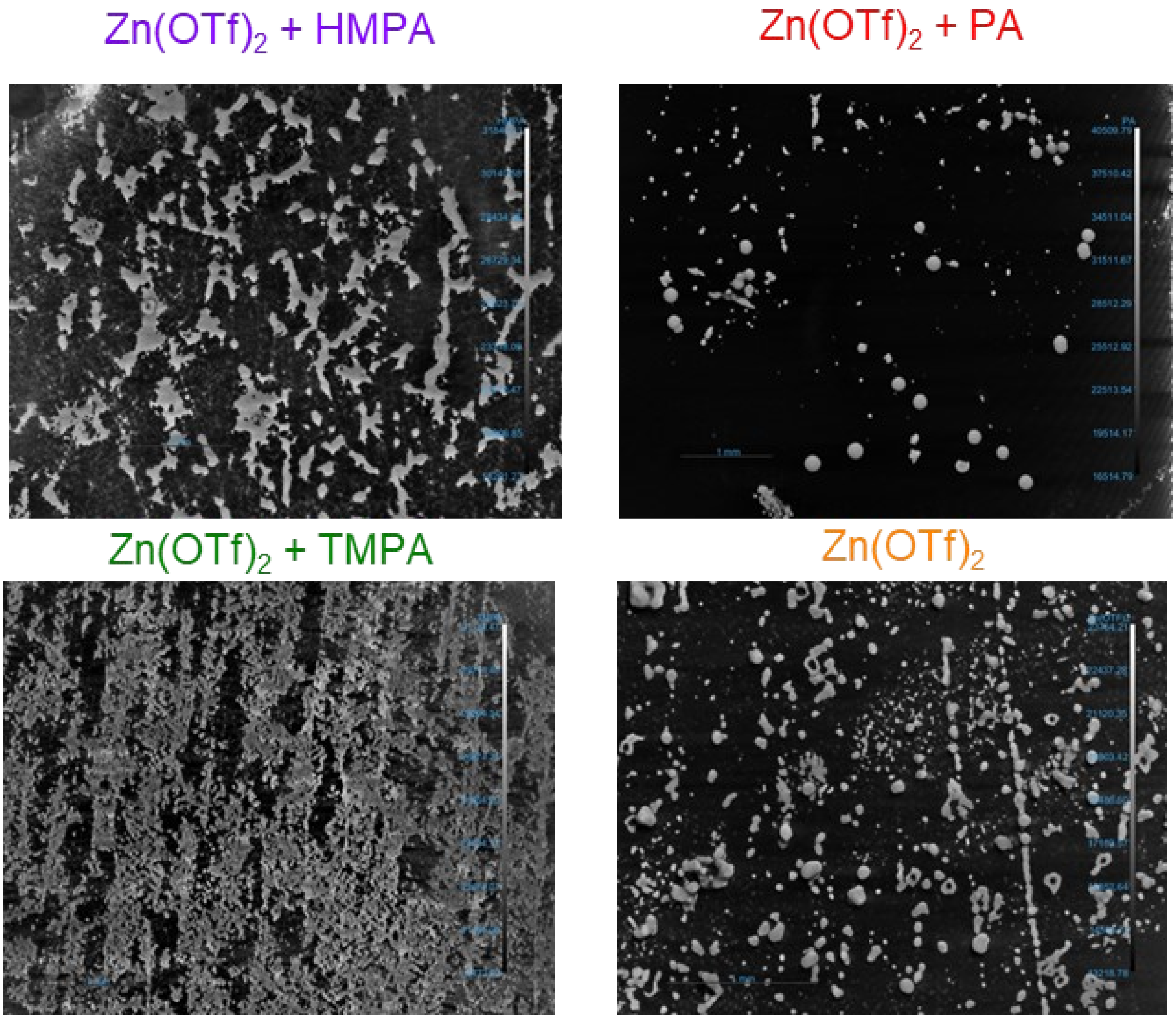

Figure S12. Zoomed in images of deposition from Xray-CT for all the electrolytes showing deposition at 2.5 mA/cm^2 (5 mAh/cm^2). The scale bar is 1 mm.

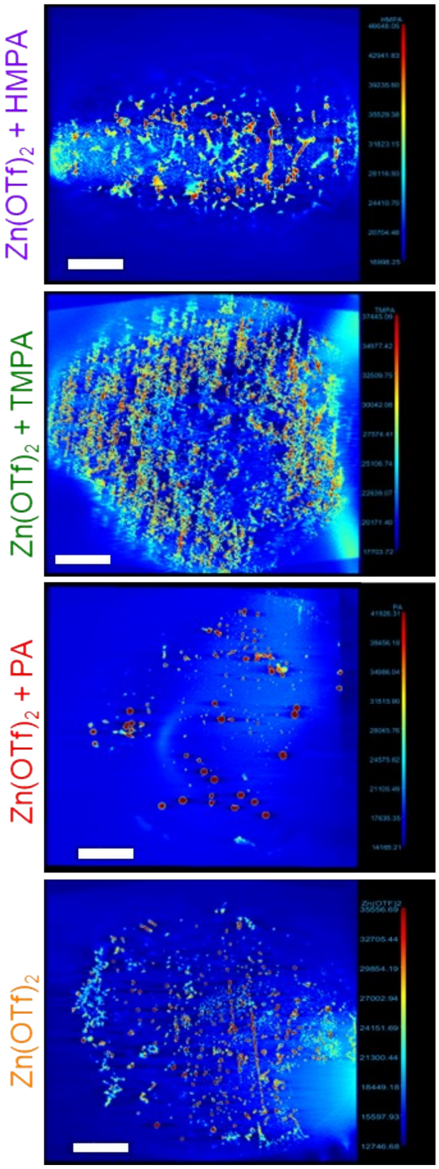

Figure S13. Contour plots for all the electrolytes showing deposition porosity at 2.5 mA/cm^2 (5 mAh/cm^2) from X-ray CT. The scale bar is 1 mm.

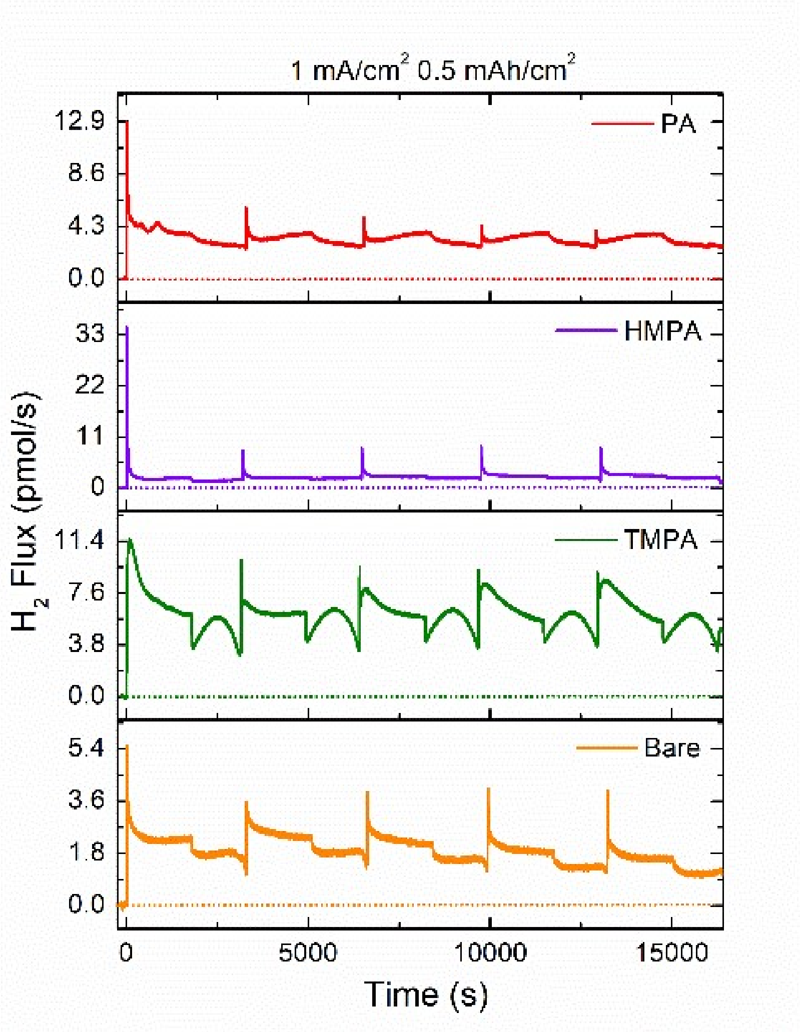

Figure S14. Electrochemical mass spectrometry (ECMS) data showing the real-time flux of H_2 gas evolved during zinc plating and stripping. The experimental setup consisted of a Cu disk (0.196 cm^2) working electrode, a Zn counter electrode, and an Ag/AgCl (1 M KCl) reference electrode. A current density of 1 mA/cm^2 was applied to achieve a plated capacity of 0.5 mAh/cm^2. A cutoff voltage of -0.4 V vs Ag/AgCl was used for stripping. The corresponding first-cycle voltage vs time profile is shown in Figure SX for visualization.

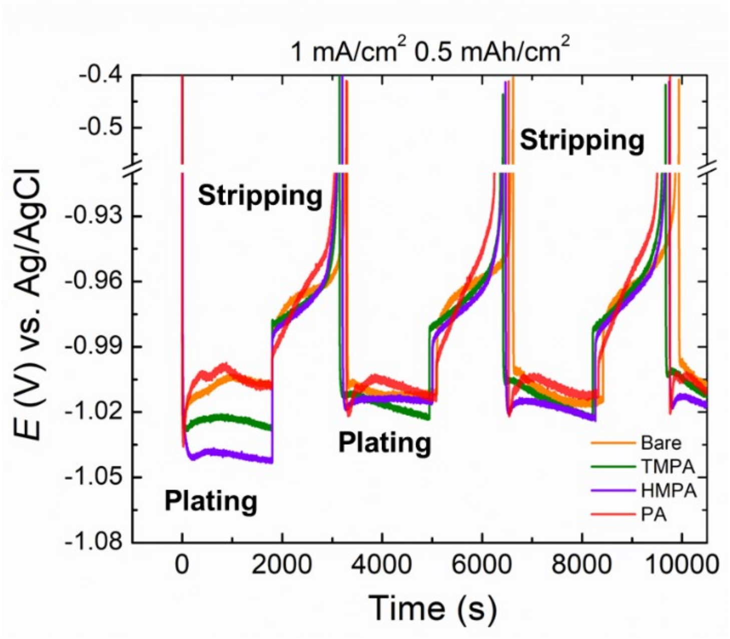

Figure S15. Typical voltage vs time recorded for the ECMS experiment presented in Figure S14.

Table S5. Calculated binding energies of the additive molecules and other ligands on Zn(002), and Zn(101) facets on electrode.

Species binding to Zn Surface	ΔE_{bind} (eV) (PBE+D3)	
	**Zn(002)**	**Zn(101)**
HMPA	-1.82	-5.21
TMPA	-1.94	-5.17
PA	-1.04	-0.99
OTf^-	-3.87	-0.73

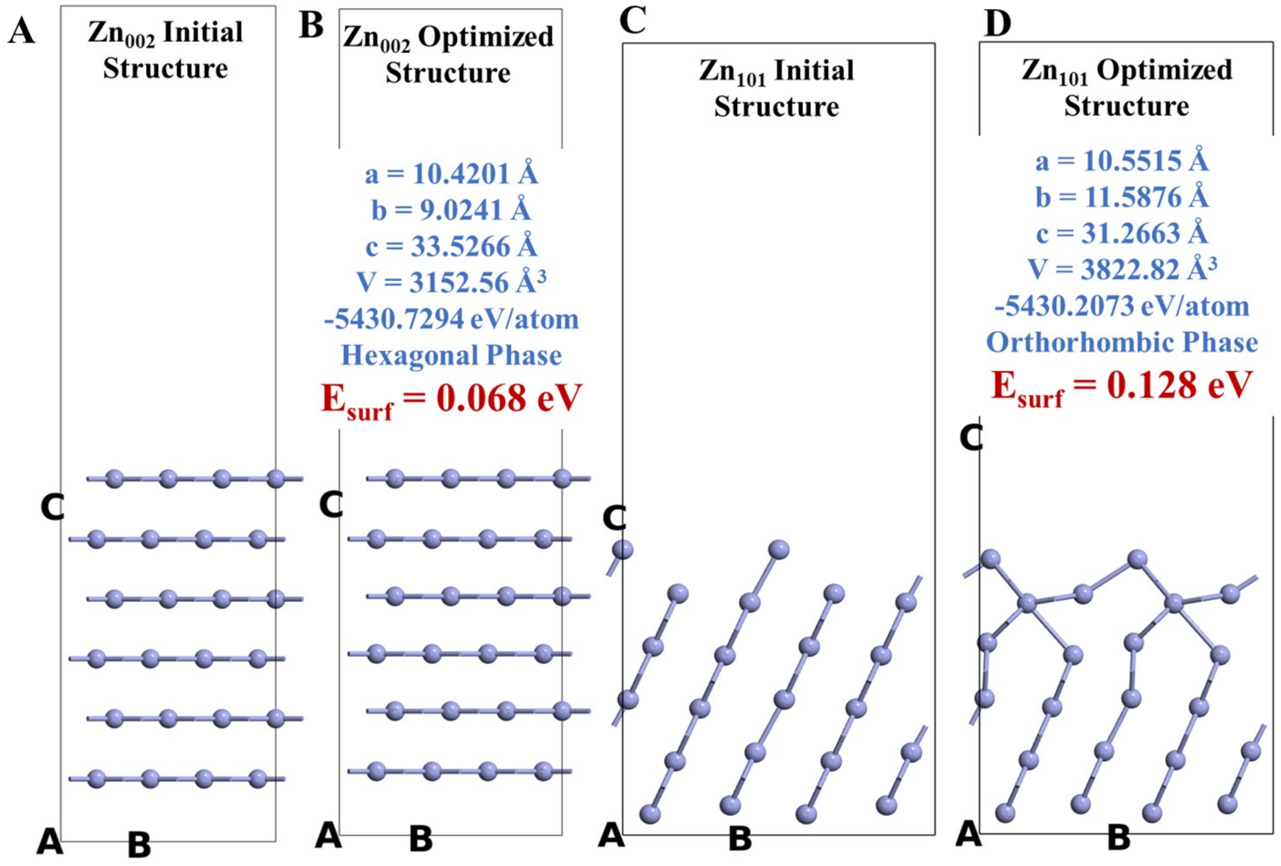

Figure S16. Initial and optimized structure of Zn(002) (A, B), and Zn(101) (C, D) surface. The lower three layers are fixed, surface energy, E_{surf} for optimized structures are mentioned. The tendency of surface Zn atoms to reconstruct in (101) facets of Zn anode is clearly visible in (d) in which the lower layers of atoms are held fixed.

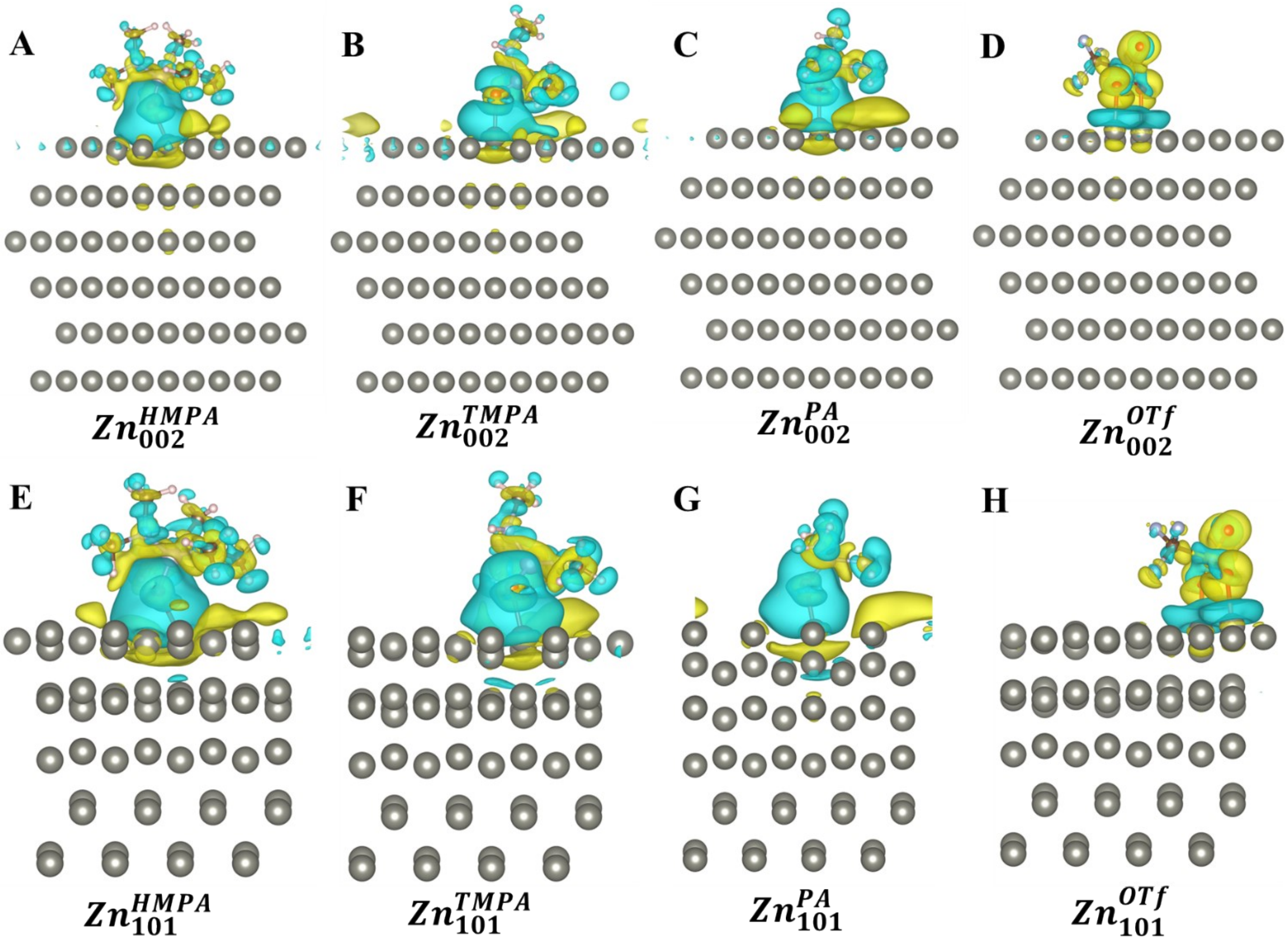

Figure S17. Differential charge density of different additives over (A-D) Zn(002), and (E-H) Zn(101) surface. Yellow, and cyan represents accumulation, and depletion, respectively. Charge depletion over Zn-O bond, wider charge density difference over Zn(101), compared to Zn(002) surface. Charge depletion over H, and accumulation over surface Zn-atom represents Zn-H interaction. Presence of charge density distribution beyond first layer of Zn atoms represents the surface reconstruction for Zn(101) surface.

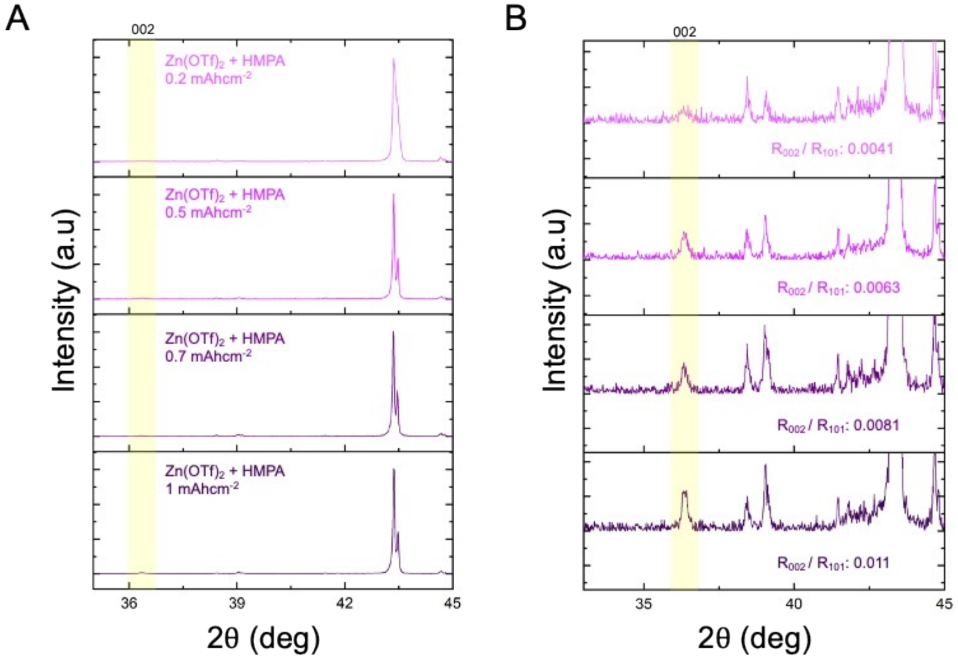

Figure S18. (A) XRD of zinc electrodeposited on a Cu foil in $Zn(OTf)_2$ + HMPA at different capacities (B) Zoomed in XRD to measure the different Zn(002) intensities at different capacities.

References

(1) Frisch, M. J.; Trucks, G. W.; Schlegel, H. B.; Scuseria, G. E.; Robb, M. A.; Cheeseman, J. R.; Scalmani, G.; Barone, V.; Petersson, G. A.; Nakatsuji, H.; Li, X.; Caricato, M.; Marenich, A. V.; Bloino, J.; Janesko, B. G.; Gomperts, R.; Mennucci, B.; Hratchian, H. P.; Ortiz, J. V.; Izmaylov, A. F.; Sonnenberg, J. L.; Williams-Young, D.; Ding, F.; Lipparini, F.; Egidi, F.; Goings, J.; Peng, B.; Petrone, A.; Henderson, T.; Ranasinghe, D.; Zakrzewski, V. G.; Gao, J.; Rega, N.; Zheng, G.; Liang, W.; Hada, M.; Ehara, M.; Toyota, K.; Fukuda, R.; Hasegawa, J.; Ishida, M.; Nakajima, T.; Honda, Y.; Kitao, O.; Nakai, H.; Vreven, T.; Throssell, K.; Montgomery, J. A., Jr.; Peralta, J. E.; Ogliaro, F.; Bearpark, M. J.; Heyd, J. J.; Brothers, E. N.; Kudin, K. N.; Staroverov, V. N.; Keith, T. A.; Kobayashi, R.; Normand, J.; Raghavachari, K.; Rendell, A. P.; Burant, J. C.; Iyengar, S. S.; Tomasi, J.; Cossi, M.; Millam, J. M.; Klene, M.; Adamo, C.; Cammi, R.; Ochterski, J. W.; Martin, R. L.; Morokuma, K.; Farkas, O.; Foresman, J. B.; Fox, D. J. Gaussian 16, Revision C.01. *Gaussian, Inc, Wallingford CT* 2016.

(2) Becke, A. D. Density-Functional Thermochemistry. III. The Role of Exact Exchange. *J Chem Phys* 1993, *98* (7), 5648–5652. https://doi.org/10.1063/1.464913.

(3) Lee, C.; Yang, W.; Parr, R. G. Development of the Colle-Salvetti Correlation-Energy Formula into a Functional of the Electron Density. *Phys Rev B* 1988, *37* (2), 785–789. https://doi.org/10.1103/PhysRevB.37.785.

(4) Vosko, S. H.; Wilk, L.; Nusair, M. Accurate Spin-Dependent Electron Liquid Correlation Energies for Local Spin Density Calculations: A Critical Analysis. *Can J Phys* 1980, *58* (8), 1200–1211. https://doi.org/10.1139/p80-159.

(5) Stephens, P. J.; Devlin, F. J.; Chabalowski, C. F.; Frisch, M. J. Ab Initio Calculation of Vibrational Absorption and Circular Dichroism Spectra Using Density Functional Force Fields. *J Phys Chem* 1994, *98* (45), 11623–11627. https://doi.org/10.1021/j100096a001.

(6) Pearson, R. G. Absolute Electronegativity and Hardness Correlated with Molecular Orbital Theory. *Proceedings of the National Academy of Sciences* 1986, *83* (22), 8440–8441. https://doi.org/10.1073/pnas.83.22.8440.

(7) Grimme, S.; Antony, J.; Ehrlich, S.; Krieg, H. A Consistent and Accurate *Ab Initio* Parametrization of Density Functional Dispersion Correction (DFT-D) for the 94 Elements H-Pu. *J Chem Phys* 2010, *132* (15). https://doi.org/10.1063/1.3382344.

(8) Zhao, Y.; Schultz, N. E.; Truhlar, D. G. Design of Density Functionals by Combining the Method of Constraint Satisfaction with Parametrization for Thermochemistry, Thermochemical Kinetics, and Noncovalent Interactions. *J Chem Theory Comput* 2006, *2* (2), 364–382. https://doi.org/10.1021/ct0502763.

(9) Marenich, A. V.; Cramer, C. J.; Truhlar, D. G. Universal Solvation Model Based on Solute Electron Density and on a Continuum Model of the Solvent Defined by the Bulk Dielectric Constant and Atomic Surface Tensions. *J Phys Chem B* 2009, *113* (18), 6378–6396. https://doi.org/10.1021/jp810292n.

(10) Smidstrup, S.; Stradi, D.; Wellendorff, J.; Khomyakov, P. A.; Vej-Hansen, U. G.; Lee, M.-E.; Ghosh, T.; Jónsson, E.; Jónsson, H.; Stokbro, K. First-Principles Green's-Function Method for Surface Calculations: A Pseudopotential Localized Basis Set Approach. *Phys Rev B* 2017, *96* (19), 195309. https://doi.org/10.1103/PhysRevB.96.195309.
